# Detection, Discrimination, and Localization of Rotor Winding Faults in Doubly Fed Induction Generators Using a Three-Layer ZSC–CASI–CADI Framework

**DOI:** 10.3390/s26010273

**Published:** 2026-01-01

**Authors:** Muhammad Shahzad Aziz, Jianzhong Zhang, Sarvarbek Ruzimov, Xu Huang, Anees Ahmad

**Affiliations:** School of Electrical Engineering, Southeast University, Nanjing 210096, China; shahzadaziz@seu.edu.cn (M.S.A.); s.k.ruzimov@seu.edu.cn (S.R.); huangxuee@seu.edu.cn (X.H.); engr.aneesahmad@seu.edu.cn (A.A.)

**Keywords:** DFIG, rotor winding faults, inter-turn short circuit fault, high resistance connection fault, zero sequence current, fault detection

## Abstract

Reliable detection of the rotor winding faults in the doubly fed induction generator (DFIG) is crucial for the resilience of the variable speed energy systems. High-resistance connection (HRC) and inter-turn short circuit (ITSC) faults cause current distortions that are remarkably similar, and the rapid rotor side dynamics and the DFIG multimode operation ability also make fault diagnosis more difficult. This paper proposes a three-layer diagnostic framework named ZSC-CASI-CADI which leverages three-phase rotor currents in conjunction with rotor zero-sequence current (ZSC) for comprehensive rotor winding fault diagnosis. Fault detection is realized through ZSC magnitude and the Cosine Angle Spread Indicator (CASI) enables the strong discrimination between HRC and ITSC faults using the dispersion of rotor current phasors from the ZSC reference. Fault localization is achieved using the Current Angle Difference Indicator (CADI), which determines the faulty rotor phase through the angular deviations in rotor currents from the ZSC. The methodology is verified with extensive simulation results to demonstrate the accurate, real-time fault detection, discrimination, and localization of DFIG rotor winding faults under different load and rotor speed conditions including sub-synchronous and super-synchronous modes. The results show that the proposed framework provides a light and effective solution for rotor winding fault monitoring of the DFIG systems.

## 1. Introduction

### 1.1. Motivation

Wind power has become an essential source of sustainable electricity through the energy transformation process in the world. Through this process, the doubly fed induction generator (DFIG) has also become common technology for variable speed wind turbines. It is inexpensive which can mainly be explained by the fact that it uses partial-rated power converters. Moreover, the DFIG provides good performance over a broad operating range, which allows active and reactive power to be independently controlled [1,2,3]. However, the DFIGs have some weaknesses as well. For example, the rotor circuits are subjected to various electrical and mechanical dynamics and are not able to resist wear in the long run. Slip ring assembly failure, thermal cycling and grid voltage variations are some of the problems which can damage the windings in the rotor. Unless addressed at an early stage, these problems can lead to severe electrical faults. The most prevalent faults are high-resistance connection (HRC) and inter-turn short circuit (ITSC) that have significant impacts on the generator health [4,5]. The common causes of HRC fault include loose connection, contact corrosion or poor thermal dissipation, which gradually cause additional resistance in the windings. Such increased resistance can lead to localized overheating, voltage imbalance and torque variation [6]. On the other hand, the main cause of the ITSC is the failure in the insulation between the winding turns, which creates a low-resistance circuit. It results in the high fault current present in the circuit and once it is left unattended, it might readily develop phase fault or ground fault [7,8]. Both the HRC and ITSC faults make current asymmetrical, although they differ in terms of their causes and patterns of development. An HRC develops slowly and the system can keep running while it is being monitored. In contrast, an ITSC develops quickly and usually requires quickly shutting down the system to avoid serious damage [9]. Because of these differences, a diagnostic system needs to do more than just detecting a fault. It should also discriminate the fault type and identify the faulty phase. This does help to ensure correct maintenance and prevent unnecessary downtime.

### 1.2. Review of the Existing Diagnostic Techniques

Much research efforts on the condition monitoring and fault diagnosis of rotor and stator faults in DFIGs as well as a variety of other electrical machines have been carried out, suggesting various diagnostic techniques [10,11]. These diagnostic techniques are used to evaluate various electrical and mechanical signals such as current [12], vibration [13], transient leakage flux [14], magnetic air gap flux [15], power [16], torque [17], speed [18,19], and temperature variations [20]. In general, the existing diagnostic techniques can be categorized as signal-based, model-based, and data-driven based. Depending on the available computational power, the suitability of available models and the characteristics of the available data, each category will have its own set of benefits and difficulties.

Signal-based diagnostics are often chosen for industrial applications because they are non-intrusive diagnostic techniques and can be used without changes to the operating parameters of the machines. Motor Current Signature Analysis (MCSA) techniques are the most widely used signal-based techniques that examine the current signals by using signal processing tools like Fast Fourier Transform (FFT) [21], Discrete Wavelet Transform (DWT) [22], Hilbert–Huang Transform [23], and Empirical Mode Decomposition (EMD) [17], and help to identify the anomalies related to stator and rotor winding faults in induction machines. These anomalies are then compared to the known operational benchmarks hence leading to the detection of the machine faults [24]. However, mixing of fault-related harmonic content found in machine signals with that found in healthy winding condition or their suppression by the control system can reduce the effectiveness of MCSA techniques in realistic settings. The tools used in these techniques are often too computationally expensive for real-time fault detection. Also, the fault location is not identified by these techniques [25].

Controller signals-based technique uses internal control signals such as rotor position, reference errors, and internal feedback which are useful for increasing the accuracy of fault detection when external electrical signatures in MCSA are unclear or conditions are fluctuating. Yet, these internal signals usually do not indicate the exact faulty phase, but rather indicate the general condition of the system as a whole [25]. Consequently, more analytical procedures are still necessary to identify the specific faulty phase for specific maintenance.

Other signal-based techniques, like Park’s Vector and Sequence Component analysis, also help identify current imbalances and phase distortions caused by the faults. Negative-sequence current component (NSCC) can detect stator winding ITSC faults in inverter-fed induction motors [26]. Zero-Sequence Voltage Component (ZSVC) is a sensitive indicator of winding faults and can effectively detect ITSC and HRC faults and identify their location in wye-connected stators of induction machines including Permanent-Magnet Synchronous Machines (PMSMs) and DFIGs [11,27,28]. For delta-connected machines, however, measuring zero-sequence voltage directly is not feasible. In this case, diagnostic techniques focus on the Zero-Sequence Current (ZSC), which circulates within the closed-loop winding [10,29]. ZSC is useful for detecting ITSC and HRC faults and identifying their location in delta-connected stators of induction machines [30,31].

Model-based diagnostics take a different approach, relying on mathematical models of machine behaviour. Faults are detected when discrepancies between measured values and model predictions exceed a certain threshold. This can involve techniques like parameter estimation and state observers [32]. These methods are effective at tracking changes in machine parameters like resistance or inductance caused by the faults [33]. However, their primary drawback is their reliance on accurate models. Changes in load or temperature can cause the model to diverge from actual conditions, leading to false alarms [34].

With the advent of more powerful processors and the availability of larger datasets, data-driven methods and artificial intelligence (AI) techniques are becoming more common. Approaches like Artificial Neural Networks (ANNs) [35], Support Vector Machines (SVMs) [36], and Fuzzy-Logic systems [37] are now used to detect and classify faults based on learned patterns. More advanced deep learning models, such as Convolutional Neural Networks (CNNs) and Recurrent Neural Networks (RNNs), have shown impressive accuracy in diagnosing the faults in machines [38]. While these techniques hold significant promise, they require large and high-quality datasets, which can be challenging and costly in data gathering, especially for rare or severe faults.

### 1.3. Challenges in Existing Diagnostic Techniques

Upon reviewing the literature on DFIG fault detection, a few persistent challenges emerge as follows:

- Many studies, particularly those involving ZSC and ZSV analysis, focus on stator faults or are designed for general induction motors where the supply frequency remains constant [27,29,31,39]. However, the DFIG rotor operates at variable slip and hence a variable frequency which distorts or even completely masks rotor fault signatures due to the influence of the converters’ control loops [40]. This makes rotor fault detection significantly more difficult.
- DFIG rotors are typically delta-connected, which limits the applicability of methods like ZSV analysis, which require a neutral point. Efforts to create artificial neutrals, such as capacitive networks [39], increase complexity and cost, making them impractical for converter-fed rotor circuits.
- While some studies have tried to detect rotor faults using rotor currents or search-coil voltages [12,41], only a few have successfully distinguished between HRC and ITSC faults. Many attempts to do so rely on complex time–frequency analyses. The way these faults affect the phase angles of rotor currents relative to ZSC has not been fully exploited as a diagnostic tool in variable frequency DFIG rotor windings.
- Many diagnostic systems can detect the presence of a fault but cannot locate the exact rotor phase involved. In the absence of such localization, maintenance staff is forced to do inspections across all phases, thus increasing the duration of downtime and hence increasing the operational costs.

These challenges highlight the need for a diagnostic framework which is simple, reliable and computationally light, especially to deal with rotor winding faults in DFIG. A satisfactory framework would include detection, discrimination and localization in order to be effective where a large additional hardware and demanding signal processing are not required.

### 1.4. Key Contributions

In order to address these challenges, a new three-layer diagnostic framework called the ZSC-CASI-CADI framework is proposed in the present study. The main contributions of this work are as follows:

- This study introduces an organized diagnostic framework that can diagnose DFIG rotor winding faults under super-synchronous operation and dynamic low-frequency rotor conditions, which are often ignored in the existing literature. The framework systematically progresses from fault detection to fault discrimination and finally to faulty phase localization. This continuous point-to-point approach provides a complete diagnostic solution going beyond simple detection of faults to give the opportunity of real actionable insight for maintenance.
- This study presents two new and computationally reasonable metrics developed from the phase angles of three rotor currents and the zero-sequence current (ZSC). The first one, Cosine Angle Spread Indicator (CASI), supports healthy discrimination between ITSC and HRC faults. The second one, Current Angle Difference Indicator (CADI) supports accurate fault localization of the specific faulty phase under both sub-synchronous and super-synchronous operating modes.
- The entire framework has been specially designed to take into account the special challenges found in the delta-connected, variable-frequency, converters-fed DFIG rotor system. Using the ZSC as a key diagnostic signal, it overcomes the limitations of stator-based or neutral point-based techniques.

### 1.5. Paper Organization

Further parts of this paper are organized as follows: In Section 2, models of DFIG with rotor winding ITSC and HRC faults are presented. In Section 3, analytical evaluation of the models is conducted, in which fault indicators are defined and the proposed three-layer ZSC–CASI–CADI framework for fault detection is described. Section 4 performs simulation studies and describes the results obtained. Finally, Section 5 offers the conclusions drawn from the study.

## 2. Modeling of DIFG

### 2.1. DFIG Configuration

**Figure 1:**
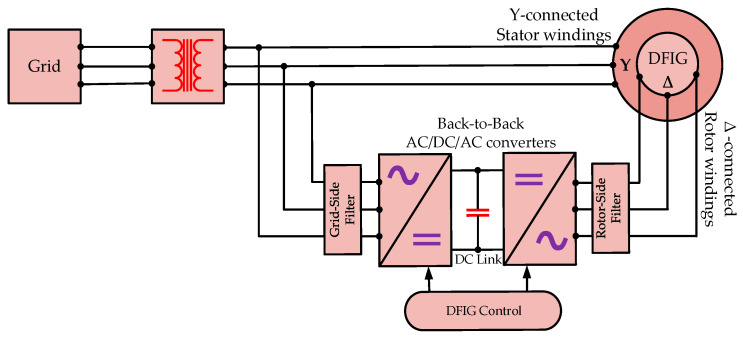
The DFIG system of Vestas V90 with Y-∆ windings configuration.

The DFIG used in wind turbines has three-phase stator windings directly connected to the power grid. The rotor has three-phase windings independent of the stator and is connected to the grid with AC/DC/AC converters by the slip rings. This topology makes it possible to control the frequencies and power flows. In order to achieve voltage compatibility between generator end and the grid, a three-phase transformer is placed on the grid side of the system. In this configuration, the system can adapt dynamically to the changing operating conditions for the purposes of optimal performance. The entire operation of the generator is controlled by an extensive control system, through which the generator adjusts its response to wind speed variations and changing grid demands, which makes the DFIG a flexible and efficient system for renewable generation. Figure 1 shows the topology of a typical commercial Vestas V90 DFIG system.

### 2.2. DFIG Rotor Winding Faults

**Figure 2:**
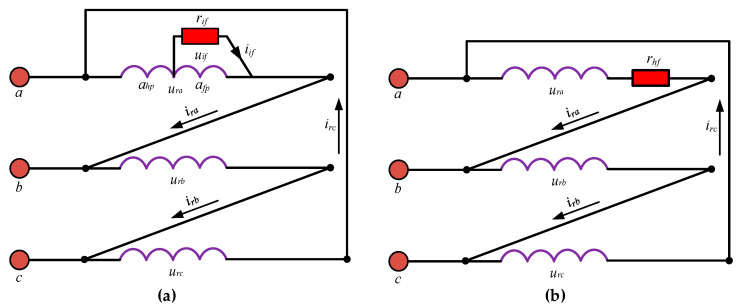
Schematic of faults in phase-a of the ∆-connected rotor winding of DFIG: (**a**) ITSC; (**b**) HRC.

The most important faults of DFIG rotor windings are HRC and ITSC. An ITSC fault is an insulation failure in one of the phases of rotor windings of the DFIG. Figure 2a shows the schematic of ITSC fault in phase-a of the delta-connected rotor winding. *r_if_*, *i_if_*, and *u_if_* are the fault resistance, fault current, and fault voltage due to the occurrence of ITSC fault, respectively. When ITSC fault happens, the faulty phase-a divides into two parts and the rotor winding of the generator has four phases including one phase due to the fault. So, *a_hp_* and *a_fp_* are the healthy and faulty part of the faulty phase-a, respectively. An increased number of faulty turns decreases the fault resistance *r_if_* and hence the overall resistance of the faulty phase.

An HRC fault in any phase of the delta-connected rotor winding of a DFIG is due to the high resistance *r_hf_* caused by the factors such as loose joints, corrosion of joints, or abnormal temperature increase within the windings. Figure 2b shows the schematic of the HRC fault in phase-a of the delta-connected rotor winding. As *r_hf_* increases, the phase current *i_ra_* through the faulty phase-a decreases which reduces the current magnitude and disturbs the current balance among the rotor phases.

The additional dynamics appear in the DFIG system due to the change in the faulty phase parameters. A detailed study of these dynamics can be helpful as a diagnostic tool for determining faults in rotor windings of the DFIG.

### 2.3. DFIG Model with Rotor ITSC

To include an ITSC fault in any phase of the rotor, a DFIG model based on the abc-reference frame is used. Referring to Figure 2a, when ITSC happens, DFIG has an extra phase represented by the voltage *u_if_* and the resistance *r_if_* in parallel with the faulty part *a_fp_* of the phase. The extent of the ITSC fault is described by the parameter *µ*, which is simply the ratio of shorted turns *n_s_* and the total turns *n_t_* in a phase, i.e., *n_s_*/*n_t_*. The following voltage equations are therefore applicable for a DFIG that has an ITSC fault in phase-a of the rotor winding:

(1) $$\left[\begin{array}{c} U_{sabc}\\ \begin{array}{l} U_{rabc}\\ u_{if} \end{array} \end{array}\right]=\left[\begin{array}{ccc} R_{s} & 0_{3\times 3} & 0_{3\times 1}\\ 0_{3\times 3} & R_{rf} & 0_{3\times 1}\\ 0_{1\times 3} & 0_{1\times 3} & \mu r_{r} \end{array}\right]\left[\begin{array}{c} i_{sabc}\\ i_{rabc}\\ i_{if} \end{array}\right]+\frac{d}{dt}\left[\begin{array}{c} \phi_{sabc}\\ \phi_{rabc}\\ \phi_{if} \end{array}\right]$$

(2) $$\left[\begin{array}{c} \phi_{sabc}\\ \phi_{rabc}\\ \phi_{if} \end{array}\right]=\left[\begin{array}{ccc} L_{ss} & L_{sr} & \mu L_{1}\\ L_{rs} & L_{rr} & \mu L_{2}\\ \mu L_{1}^{T} & \mu L_{2}^{T} & \mu^{2}L_{r} \end{array}\right]\left[\begin{array}{c} i_{sabc}\\ i_{rabc}\\ i_{if} \end{array}\right]$$

where *U_sabc_* and *U_rabc_* are three-phase stator and rotor voltages, respectively; *i_sabc_* and *i_rabc_* are three-phase stator and rotor currents, respectively; *ϕ_sabc_* and *ϕ_rabc_* are the stator and rotor magnetic fluxes, respectively; *R_s_* and *R_rif_* are three-phase stator and rotor resistance under fault, respectively. *u_if_*, *i_if_*, and *ϕ_if_* are voltage, current, and magnetic flux due to ITSC fault, respectively. The following equations further explain these terms.

$$\begin{array}{l} \left[U_{sabc}\right]=\left[\begin{array}{ccc} u_{sa} & u_{sb} & u_{sc} \end{array}\right];\left[U_{rabc}\right]=\left[\begin{array}{ccc} u_{ra} & u_{rb} & u_{rc} \end{array}\right];\left[i_{sabc}\right]=\left[\begin{array}{ccc} i_{sa} & i_{sb} & i_{sc} \end{array}\right];\\ \left[i_{rabc}\right]=\left[\begin{array}{ccc} i_{ra} & i_{rb} & i_{rc} \end{array}\right];\left[\phi_{sabc}\right]=\left[\begin{array}{ccc} \phi_{sa} & \phi_{sb} & \phi_{sc} \end{array}\right];\left[\phi_{rabc}\right]=\left[\begin{array}{ccc} \phi_{ra} & \phi_{rb} & \phi_{rc} \end{array}\right]; \end{array}$$

ITSC fault raises an electrical asymmetry due to internal winding damage. This fault has a direct impact on the magnetic circuit of the machine which causes a change in inductance matrices as well as resistance matrices. Therefore, the modified matrices are written as follows:

$$R_{s}=\left[\begin{array}{ccc} r_{s} & 0 & 0\\ 0 & r_{s} & 0\\ 0 & 0 & r_{s} \end{array}\right];\;L_{ss}=\left[\begin{array}{ccc} L_{s} & -L_{m} & -L_{m}\\ -L_{m} & L_{s} & -L_{m}\\ -L_{m} & -L_{m} & L_{s} \end{array}\right];$$

$$R_{rif}=\left[\begin{array}{ccc} (1-\mu )r_{r} & 0 & 0\\ 0 & r_{r} & 0\\ 0 & 0 & r_{r} \end{array}\right];\;L_{rr}=\left[\begin{array}{ccc} {\left(1-\mu \right)}^{2}L_{r} & -(1-\mu )L_{rm} & -(1-\mu )L_{rm}\\ -(1-\mu )L_{rm} & L_{r} & L_{mr}\\ -(1-\mu )L_{rm} & L_{rm} & L_{r} \end{array}\right];$$

$$L_{sr}=L_{rs}^{T}=M_{r}\left[\begin{array}{ccc} (1-\mu )\cos\theta_{r} & \cos(\theta_{r}-\frac{2\pi }{3}) & \cos(\theta_{r}+\frac{2\pi }{3})\\ \left(1-\mu )\cos(\theta_{r}+\frac{2\pi }{3}\right) & \cos\theta_{r} & \cos(\theta_{r}-\frac{2\pi }{3})\\ \left(1-\mu )\cos(\theta_{r}-\frac{2\pi }{3}\right) & \cos(\theta_{r}+\frac{2\pi }{3}) & \cos\theta_{r} \end{array}\right]$$

$$L_{1}={\left[\begin{array}{ccc} (1-\mu )L_{r} & -\frac{1}{2}M_{r} & -\frac{1}{2}M_{r} \end{array}\right]}^{T};\;L_{2}={\left[\begin{array}{ccc} \cos\theta_{r} & \cos(\theta_{r}-\frac{2\pi }{3}) & \cos(\theta_{r}+\frac{2\pi }{3}) \end{array}\right]}^{T}$$

where *L_sm_* = 1/2*M_s_*, and *L_rm_* = 1/2*M_r_*; *M_s_*, *L_s_*, and *r_s_* represent stator side quantities, namely stator mutual inductance, stator total inductance, and per-phase stator resistance, respectively. *M_r_*, *L_r_*, and *r_r_*_,_ respectively, represent the same quantities for the rotor side. The following equation models the electromagnetic torque of the DFIG with ITSC fault in one phase:

(3) $$T_{e}=P\left(i_{s}^{T}\frac{\partial L_{sr}}{\partial \theta_{r}}i_{r}+\mu f_{x}^{T}i_{f}\frac{\partial L_{sr}}{\partial \theta_{r}}i_{r}\right)$$

And the mechanical motion is modeled by the following equation:

(4) $$T_{m}=T_{e}-\left(\frac{J}{n_{p}}\frac{d\omega_{e}}{dt}+\frac{D\omega_{e}}{n_{p}}\right)$$

where *T_e_* and *T_m_* represent electromagnetic and mechanical torques of the machine, respectively. *D* is the damping coefficient while *J* represents the inertia of the machine. Further, *n_p_* is the number of pole pairs and *ω_e_* is the electrical angular velocity.

### 2.4. DFIG Model with Rotor HRC

To include an HRC fault in any phase of the rotor, an additional resistance *r_hf_* is added in the faulty phase using the DFIG model in the abc-reference frame. Referring to Figure 2b, when HRC happens, it does not create any extra phase. The extent of the HRC fault depends on the value of the added resistance *r_hf_*. The following voltage equations are therefore applicable for a DFIG that has an HRC fault in phase-a of its rotor winding:

(5) $$\left[\begin{array}{c} U_{sabc}\\ U_{rabc} \end{array}\right]=\left[\begin{array}{cc} R_{s} & 0_{3\times 3}\\ 0_{3\times 3} & R_{rhf} \end{array}\right]\left[\begin{array}{c} i_{sabc}\\ i_{rabc} \end{array}\right]+\frac{d}{dt}\left[\begin{array}{c} \phi_{sabc}\\ \phi_{rabc} \end{array}\right]$$

(6) $$\left[\begin{array}{c} \phi_{sabc}\\ \phi_{rabc} \end{array}\right]=\left[\begin{array}{cc} L_{ss} & L_{sr}\\ L_{rs} & L_{rr} \end{array}\right]\left[\begin{array}{c} i_{sabc}\\ i_{rabc} \end{array}\right]$$

HRC fault raises an electrical asymmetry due to external path resistance. This fault has no direct impact on the magnetic circuit of the machine and causes no change in inductance matrices. It only impacts the resistance matrices. Therefore, the matrices are written as follows:

$$\;R_{rhf}=\left[\begin{array}{ccc} r_{r}+r_{hf} & 0 & 0\\ 0 & r_{r} & 0\\ 0 & 0 & r_{r} \end{array}\right];\;L_{ss}=\left[\begin{array}{ccc} L_{s} & -L_{sm} & -L_{sm}\\ -L_{sm} & L_{s} & -L_{sm}\\ -L_{sm} & -L_{sm} & L_{s} \end{array}\right];\;L_{rr}=\left[\begin{array}{ccc} L_{r} & -L_{rm} & -L_{rm}\\ -L_{rm} & L_{r} & -L_{mr}\\ -L_{rm} & -L_{rm} & L_{r} \end{array}\right];$$

$$L_{sr}=L_{rs}^{T}=M_{r}\left[\begin{array}{ccc} \cos\theta_{r} & \cos(\theta_{r}-\frac{2\pi }{3}) & \cos(\theta_{r}+\frac{2\pi }{3})\\ \cos(\theta_{r}+\frac{2\pi }{3}) & \cos\theta_{r} & \cos(\theta_{r}-\frac{2\pi }{3})\\ \cos(\theta_{r}-\frac{2\pi }{3}) & \cos(\theta_{r}+\frac{2\pi }{3}) & \cos\theta_{r} \end{array}\right]$$

where *L_sm_* = 1/2*M_s_*, and *L_rm_* = 1/2*M_r_*. The electromagnetic torque of the DFIG can be expressed as follows:

(7) $$T_{e}=P\left(i_{s}^{T}\frac{\partial L_{sr}}{\partial \theta_{r}}i_{r}\right)$$

## 3. Methodology

The methodology section presents the proposed diagnostic strategy developed to identify and distinguish between ITSC and HRC faults in the rotor windings of a DFIG. The stator windings of DFIG under study is Y-connected and the rotor windings are ∆-connected. The methodology is principally based on the analysis of ZSC but introduces an extended framework based on analytical evaluation that enables separate recognition and classification of ITSC and HRC faults within the rotor windings.

### 3.1. ZSC Signal in ∆-Connected Rotor Windings

The back-to-back power converters supply the ∆-connected rotor windings. In a healthy rotor windings scenario, equal currents flow through all the three phases and maintain the symmetry. The algebraic sum of these three equal currents is zero which means that the ZSC does not flow in the rotor windings. This scenario is given by (8).

(8) $$i_{ra}+i_{rb}+i_{rc}=0\;\mathrm{and}\;i_{z}=0$$

where *i_z_* represents ZSC component in rotor windings. Upon the occurrence of faults in rotor windings, currents flowing through three phases are unequal and lose their symmetry. The algebraic sum of rotor currents no longer equals zero. Due to this asymmetry, ZSC circulates within the branches of the delta-connected rotor windings. This scenario is given by (9).

(9) $$i_{ra}+i_{rb}+i_{rc}=i_{z}\;\mathrm{and}\;i_{z}>0$$

**Figure 3:**
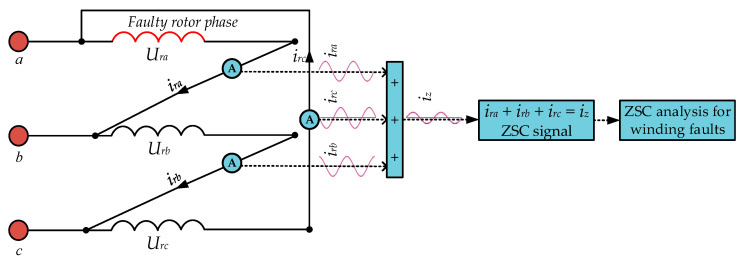
Measurement of ZSC signal in DFIG ∆-connected rotor windings.

The ZSC signal in (9) contains information about the rotor winding faults. However, this signal can be obtained through three current sensors because ZSC circulates only within the delta-connected rotor branches. Figure 3 shows the measurement of ZSC signal under rotor winding faults.

This measurement approach can enhance the fault diagnosis efficiency because it can directly extract the ZSC signal from the rotor circuit. It also improves the level of accuracy with which the exact faulty phase can be identified, since it monitors the current in each phase separately within the rotor windings. The implementation of this approach is very simple and, by using conventional measurement instrumentation, it can be added to the existing, real-time monitoring schemes with a little change in the system configuration.

#### 3.1.1. ZSC Signal Under ITSC

Application of Kirchhoff’s Voltage Law (KVL) to the ∆-connected rotor windings gives a situation where three-phase rotor voltages add up to zero. Therefore, referring to Figure 3, the following relationship is obtained:

(10) $$u_{ra}+u_{rb}+u_{rc}=0$$

Substituting *u_ra_*, *u_rb_*, and *u_rc_* from (1) into (10) and neglecting the derivative terms, the following simplified equation is given:

(11) $$0=(i_{ra}+i_{rb}+i_{rc})r_{r}-\mu i_{if}r_{r}-[\mu L_{r}+\frac{1}{2}\mu M_{r}]\frac{di_{if}}{dt}$$

Substituting (8) into (11), the following expression for ZSC is given:

(12) $$i_{z}=\frac{1}{r_{r}}[\mu i_{if}r_{r}+\mu (L_{r}+\frac{1}{2}M_{r})\frac{di_{if}}{dt}]$$

where *i_z_* is the ZSC under ITSC fault. It is shown from (12) that the ZSC reduces to zero as *i_if_* is ideally zero under healthy rotor windings scenario. Yet, *i_if_* current flows inside the windings and its measurement cannot be available. So, it is better to eliminate *i_if_* from (12). By subtracting *u_if_* from *u_ra_* in (1) to solve for *i_if_*, the following is given:

(13) $$i_{if}=\frac{\mu i_{ra}r_{r}}{r_{if}+(1-\mu )r_{r}}$$

It is obviously shown from (13) that the initial phase offset (IPO) of fault current *i_if_* is the same as that of the phase-a current *i_ra_* with ITSC fault. ZSC signal may contain harmonic components due to the power converter in rotor side and grid distortions in stator side, but the fundamental frequency component (FFC) is the most prominent and, therefore, highly sensitive to winding faults. Therefore, by considering only FFC of ZSC signal and same IPO for both *i_ra_* and *i_if_*, they can be expressed as follows:

(14) $$\begin{array}{l} i_{ra}=I_{ra}\sin(\theta +\theta_{ira})\\ i_{if}=I_{if}\sin(\theta +\theta_{ira}) \end{array}$$

where *I_ra_* and *I_if_* are the FFC magnitudes of *i_ra_* and *i_if_* signals, respectively. *θ_ira_* is IPO of the phase-a current *i_ra_*. Combining (13) and (14), and substituting them into (12), the following is yielded:

(15) $$i_{iz}=\frac{\mu^{2}}{r_{r}(r_{if}+(1-\mu )r_{r})}[r_{r}^{2}I_{ra}\sin(\theta +\theta_{ira})+r_{r}(L_{r}+\frac{1}{2}M_{r})\frac{d}{dt}(I_{ra}\sin(\theta +\theta_{ira})]$$

Only the FFC magnitudes of *i_ra_* and *i_if_* signals in (13) are considered because they have the same IPO and all other quantities are constant. Then, combining (13) and (15) results in the following expression:

(16) $$i_{iz}=\frac{\mu I_{if}}{r_{r}}[\sqrt{r_{r}^{2}+\omega_{r}^{2}{\left(L_{r}+\frac{1}{2}M_{r}\right)}^{2}}(\sin(\theta +\theta_{ira}+\delta )]$$

*i_iz_* can be given with the magnitude and a phase angle as follows:

(17) $$i_{iz}=I_{z}\sin(\theta +\theta_{z})$$

where *I_z_* and *θ_z_* are the FFC magnitude and IPO of ZSC signal, respectively, and they are given as follows:

(18) $$\left\{\begin{array}{l} I_{z}=\frac{\mu I_{if}}{r_{r}}[\sqrt{r_{r}^{2}+\omega_{r}^{2}{\left(L_{r}+\frac{1}{2}M_{r}\right)}^{2}}]\\ \theta_{z}=\theta_{ira}+\delta \\ \delta =\tan^{-1}(\omega_{r}(L_{r}+\frac{1}{2}M_{r})/r_{r}) \end{array}\right.$$

where *δ* represent the phase shift caused by the fault and can be estimated through the difference between IPOs of the FFCs in ZSC and *i_ar_* signals in this case where the ITSC is in phase-a.

#### 3.1.2. ZSC Signal Under HRC

The application of KVL to the delta-connected rotor windings gives (10). Substituting *u_ra_*, *u_rb_*, and *u_rc_* from (5) into (10) under HRC fault, the following equation is given:

(19) $$0=(i_{ra}+i_{rb}+i_{rc})r_{r}+i_{ra}r_{hf}+(L_{r}+3M_{r})[\frac{d}{dt}(i_{ra}+i_{rb}+i_{rc})]$$

Substituting (8) into (19) and ignoring derivative terms, the following expression is given for ZSC signal under HRC fault:

(20) $$i_{z}=-\frac{i_{ra}r_{hr}}{r_{r}}$$

where *i_z_* is the ZSC under HRC fault. It is obvious from (20) that the ZSC reduces to zero as *r_hr_* is ideally zero under a healthy rotor windings scenario. Considering only FFC of ZSC signal and substituting (14) into (20), the following is given:

(21) $$i_{hz}=-\frac{I_{ra}r_{hf}}{r_{r}}[\sin(\theta +\theta_{ira})]=\frac{I_{ra}r_{hf}}{r_{r}}[\sin(\theta +\theta_{ira}+\pi )]$$

Because *i_hz_* is composed of the magnitude and a phase angle, the following expression is obtained:

(22) $$\left\{\begin{array}{l} I_{z}=\frac{I_{ra}r_{hf}}{r_{r}}\\ \theta_{z}=\theta_{ira}+\pi \end{array}\right.$$

where *I_z_* and *θ_z_* are the FFC magnitude and IPO of ZSC signal under HRC fault, respectively. π represents the phase shift caused by the HRC fault. It shows that the HRC fault introduces the π shift in the faulty phase current where the HRC fault is in phase-a in this case.

### 3.2. ZSC as Fault Detector

The analytical evaluation shows that the FFC magnitude of ZSC signal (*I_z_*) mainly depends on the level of fault severity in ITSC and HRC faults as it can be observed from (18) and (22). Therefore, the FFC magnitude of ZSC signal serves as the rotor winding fault detector in the first layer of fault diagnostic framework. A larger FFC magnitude will reflect a large level of winding fault. For a reliable detection and to avoid the triggering of false alarms due to the inherent noise effects or due to small dynamic asymmetries of the DFIG system, a threshold value of *I_z_* is used. ZSC measurement over a range of variable operating conditions can be conducted for DFIG with healthy rotor windings to determine and define the threshold value of *I_z_* for fault detection. A predetermined threshold will avoid the triggering of false fault indications and produce fault signals only when the *I_z_* exceeds the predetermined threshold *I_zth_*. Then, the rotor winding fault condition is given as follows:

(23) $$I_{z}>I_{zth}$$

The reliability of the detection layer utilizing *I_z_* is improved by using the FFT to subtract the FFC from the ZSC signal and effectively suppress the influence of the harmonics and noises on the detection layer.

#### Fault Detection Under Variable Conditions

The DFIG operates across a wide speed range from sub-synchronous to super-synchronous modes, depending on wind speed and load conditions. Therefore, the fundamental frequency on the rotor side is not constant and it changes with the DFIG slip, as given below:

(24) $$f_{r}=s\times f_{s}$$

where *s* represents the DFIG slip. To ensure reliable fault detection under all operating conditions, the fault detection layer continuously tracks the rotor frequency in (24). This allows the FFC of ZSC signal to be accurately separated and analyzed, making the fault detection robust against changes in load and speed.

### 3.3. CASI as Fault Discriminator

Although FFC of ZSC signal is effective for fault detection, it cannot discriminate between ITSC and HRC faults alone due to its sensitivity to operating conditions and level of fault severity.

It is evident in (18) that the IPO of the FFC in ZSC signal under ITSC *θ_z_* depends on the IPO of the FFC in faulty phase current *θ_ira_* and the phase shift *δ* caused by the fault. However, (22) shows that the phase shift under HRC is π which is different from *δ* under ITSC. In case of ITSC fault *δ* is much smaller than π, and is generally restricted to the range [0, π/4]. This is because ITSC influences the fluxes and, consequently, the inductances of the machine. Additionally, the three-phase current angles are also affected by both types of faults. Hence, the analysis of the IPOs of the FFCs in ZSC signal and three-phase rotor currents can serve as the fault discriminator between ITSC and HRC. Therefore, CASI is presented and is used as fault discriminator between ITSC and HRC in the second layer of fault diagnostic framework. CASI measures the angular spread of the IPOs of the FFC in three-phase rotor currents with respect to the IPO of the FFC in ZSC signal. It measures the deviation in the cosine-transformed phase differences from their mean and provides insight into the asymmetry in the rotor current signals caused by the rotor winding faults.

The CASI is defined as follows:

(25) $$\mathrm{CASI}=\frac{\mathrm{Mean}\left[\right|\cos(\Delta_{iza})|,\;|\cos(\Delta_{izb})|,\;|\cos(\Delta_{izc})|]}{\;\mathrm{STD}[|\cos(\Delta_{iza})|,\;|\cos(\Delta_{\mathrm{izb}})|,\;|\cos(\Delta_{\mathrm{izc}})|]}$$

where Mean and STD are the mean and standard deviation of the cosine-transformed phase differences, respectively; *∆_izn_* = [(*θ_zn_* − π) − *θ_irn_*] for *n* = *a*, *b*, *c*; *θ_zn_* is the IPO of the FFC in ZSC signal when fault is in phase-n, where *θ_zb_* = *θ_za_* − 2π/3 and *θ_zc_* = *θ_ira_* + 2π/3; *θ_irn_* represents IPOs of the FFC in rotor currents. (18) and (22) give *θ_z_* and *θ_irn_* is calculated using (14), where the rotor currents can be given as follows:

(26) $$i_{rn}=I_{rn}\sin(\theta +\theta_{irn})$$

where *n* = *a*, *b*, *c*; *I_rn_* represents FFC magnitudes, *θ_irn_* represents the IPOs of the FFCs in rotor three-phase currents, where *θ_irb_* = *θ_ira_* − 2π/3 and *θ_irc_* = *θ_ira_* + 2π/3.

For ITSCF fault, the values for *∆_zn_* are evaluated using (18) and (26). If ITSC fault occurs in phase-a, then we have the following:

(27) $$\begin{array}{l} \Delta_{iza}=\theta_{za}-\pi -\theta_{ira}=\theta_{ira}+\delta -\pi -\theta_{ira}=\delta -\pi \approx -\pi +\delta \\ \Delta_{izb}=\theta_{za}-\pi -\theta_{irb}=\theta_{ira}+\delta -\pi -(\theta_{ira}-\frac{2\pi }{3})\approx -\frac{\pi }{3}+\delta \\ \Delta_{izc}=\theta_{za}-\pi -\theta_{irc}=\theta_{ira}+\delta -\pi -(\theta_{ira}+\frac{2\pi }{3})\approx \frac{\pi }{3}+\delta \end{array}$$

So, for ITSC in rotor phase-a, *∆_izn_* values are −*π* + *δ*, −π/3 − *δ*, and π/3 + *δ* for rotor phases *a*, *b*, and *c*, respectively. Likewise, for fault in phase *b* and *c*, these values are given in Table 1.

For HRC fault, the values for *∆_izn_* are evaluated from (22) and (26). If HRC fault occurs in phase-a, then we have the following:

(28) $$\begin{array}{l} \Delta_{iza}=\theta_{za}-\pi -\theta_{ira}=\theta_{ira}+\pi -\pi -\theta_{ira}\approx 0\\ \Delta_{izb}=\theta_{za}-\pi -\theta_{irb}=\theta_{ira}+\pi -\pi -(\theta_{ira}-\frac{2\pi }{3})\approx \frac{2\pi }{3}\\ \Delta_{izc}=\theta_{za}-\pi -\theta_{irc}=\theta_{ira}+\pi -\pi -(\theta_{ira}+\frac{2\pi }{3})\approx -\frac{2\pi }{3} \end{array}$$

So, for HRC in rotor phase-a, *∆_izn_* values are *0*, 2π/3, and *−*2π/3 for rotor phases *a*, *b*, and *c*, respectively. Likewise, for HRC in rotor phases *b* and *c*, the values are given in Table 1.

However, in both types of faults, three-phase rotor current angles and the ZSC shift angle are affected. These angles are also affected by the DFIG operating conditions including changes in load and speed. Therefore, these factors need to be considered. ITSC fault causes large changes in rotor currents (up to π/10 = 18^0^) as compared with that caused by the HRC fault (up to π/18 = 10^0^). The shift angle *δ* (up to π/4) in ITSC fault is much smaller than the shift angle π in HRC fault. The proportional changes (1/3 of the original value) in *δ* and π are taken as π/12 and π/3, respectively. Consider that *∆θ_irn_*, *∆δ_if_*, and *∆δ_hf_* represent the change in three-phase rotor current angles, the change in shift angle *δ*, and the change in shift angle π, respectively. Then, for ITSC fault in phase-a, the modified *∆_izn_* values of (27) are given as follows:

(29) $$\begin{array}{l} \Delta_{iza}=\theta_{za}-\pi -(\theta_{ira}+\Delta \theta_{ira})\approx (-\pi -\Delta \theta_{ira})+(\delta -\Delta \delta_{if})\\ \Delta_{izb}=\theta_{za}-\pi -(\theta_{irb}+\Delta \theta_{irb})\approx (-\frac{\pi }{3}+\Delta \theta_{ira})+(\delta -\Delta \delta_{if})\\ \Delta_{izc}=\theta_{za}-\pi -(\theta_{irc}+\Delta \theta_{irc})\approx (\frac{\pi }{3}-\Delta \theta_{ira})+(\delta -\Delta \delta_{if}) \end{array}$$

So, for ITSC in rotor phase-a, the modified *∆_izn_* values are (−*π* − *∆θ_ira_*) + (*δ* − *∆δ_if_*), (−π/3 + *∆θ_ira_*) + (*δ* − *∆δ_if_*), and (π/3 − *∆θ_ira_*) + (*δ* − *∆δ_if_*) for rotor phases *a*, *b*, and *c*, respectively. Likewise, for ITSC in rotor phases *b* and *c*, the values are given in Table 1.

For HRC fault in phase-a, the modified *∆_izn_* values of (28) are given as follows:

(30) $$\begin{array}{l} \Delta_{iza}=\theta_{za}-\pi -(\theta_{ira}+\Delta \theta_{ira})\approx (-\Delta \theta_{ira}-\Delta \delta_{hf})\\ \Delta_{izb}=\theta_{za}-\pi -(\theta_{irb}+\Delta \theta_{irb})\approx (\frac{2\pi }{3}+\Delta \theta_{ira}-\Delta \delta_{hf})\\ \Delta_{izc}=\theta_{za}-\pi -(\theta_{irc}+\Delta \theta_{irc})\approx (-\frac{2\pi }{3}-\Delta \theta_{ira}-\Delta \delta_{hf}) \end{array}$$

So, for HRC in rotor phase-a, the modified *∆_izn_* values are (−*∆θ_ira_* − *∆δ_hf_*), (2π/3 + *∆θ_ira_* − *∆δ_hf_*), and (−2π/3 − *∆θ_ira_* − *∆δ_if_*) for rotor phases *a*, *b*, and *c*, respectively. Likewise, for HRC in rotor phases *b* and *c*, the values are given in Table 1. These values incorporate the effects of winding faults and the DFIG operating conditions, as shown in Table 1.

#### Values of CASI for ITSC and HRC Faults

Under the ITSC fault, by setting π/10, π/4, and π/12 for *∆θ_irn_*, δ and *∆δ_if_*, respectively, and using (25), (29), a CASI value of 2.40 is obtained for the maximum value *∆δ_if_* = π/12. If *∆δ_if_* is decreased to possible lower values, such as π/20 and π/24, CASI values are 1.99 and 1.90, respectively. Under the HRC fault, by setting π/18 and π/3 for *∆θ_irn_* and *∆δ_hf_*, respectively, and using (25), (30), a CASI value of 1.83 is obtained for the maximum value *∆δ_hf_* = π/3. If *∆δ_hf_* is decreased to possible lower values, such as π/4 and π/5, CASI values are 1.49 and 1.55, respectively. It can be observed that the CASI values are greater than 1.85 for ITSC fault and less than 1.85 for HRC fault. Hence, CASI values for fault discrimination can be given as follows:

(31) $$\begin{array}{l} \mathrm{ITSC}\;\mathrm{Fault}\text{:}\;\mathrm{CASI}\;\text{>}\;1.85\\ \mathrm{HRC}\;\mathrm{Fault}\text{:}\;\mathrm{CASI}\;\text{<}\;1.85 \end{array}$$

### 3.4. CADI as Fault Position Indicator

It is obviously shown from (18) and (22) that the IPO of FFC in ZSC signal (*θ_z_*) mainly depends on the IPO of FFC in current signal of fault-carrying phase (*θ_ira_*) and the phase shifts caused by the faults (*δ* and π). Consequently, this phase relationship among *θ_z_*, *θ_ira_* and the phase shifts can be analyzed for the identification of the faulty rotor phase. CADI is presented and is used as fault position indicator for rotor ITSC and HRC faults in the third layer of fault diagnostic framework.

Under the sub-synchronous mode, for ITSC fault, the CADI is defined as the difference between IPO of the FFC in ZSC signal and the IPO of the FFC in one of the rotor three-phase currents. Using (18) and (26), the CADI is given as follows:

(32) $$CADI_{izn}=\left|\theta_{zn}-\theta_{irn}\right|=\left|\theta_{irnf}+\delta -\theta_{irn}\right|=\left|\delta \right|+\left|\theta_{irnf}-\theta_{irn}\right|$$

where *n* = *a*, *b*, *c*; *CADI_izn_* indicates *CADI* values under ITSC fault; *θ_irnf_* is the IPO of the FFC in faulty phase current. Using (26), (29) and (32), *CADI_izn_* for ITSC in phase-a is given as follows:

(33) $$\begin{array}{l} CADI_{iza}=\left|\delta -\Delta \delta_{if}\right|+\left|\theta_{ira}-(\theta_{ira}+\Delta \theta_{ira})\right|\approx \left|\delta -\Delta \delta_{if}+\Delta \theta_{ira}\right|\\ CADI_{izb}=\left|\delta -\Delta \delta_{if}\right|+\left|\theta_{ira}-\theta_{irb}\right|=\left|\delta -\Delta \delta_{if}\right|+\left|\theta_{ira}-(\theta_{ira}-2\pi /3-\Delta \theta_{ira})\right|\approx \left|\delta -\Delta \delta_{if}+2\pi /3+\Delta \theta_{ira}\right|\\ CADI_{izc}=\left|\delta -\Delta \delta_{if}\right|+\left|\theta_{ira}-\theta_{irc}\right|=\left|\delta -\Delta \delta_{if}\right|+\left|\theta_{ira}-(\theta_{ira}+2\pi /3+\Delta \theta_{ira})\right|\approx \left|\delta -\Delta \delta_{if}+2\pi /3+\Delta \theta_{ira}\right| \end{array}$$

So, for ITSC in rotor phase-a, *CADI_izn_* values are |*δ* − *∆δ_if_* + *∆θ_ira_*|, |*δ* − *∆δ_if_* + 2π/3 + *∆θ_ira_*|, and |*δ* − *∆δ_if_* + 2π/3 + *∆θ_ira_*| for rotor phases *a*, *b*, and *c*, respectively. Likewise, for ITSC in rotor phases *b* and *c*, the values are given in Table 2. It can be observed from Table 2 that *CADI_izn_* has the smallest value for the faulty rotor phase when ITSC fault occurs in one of the rotor three phases under the sub-synchronous mode where CADI is the difference between IPOs of FFCs in ZSC signal and the three-phase current signals (*θ_zn_* − *θ_irn_*).

However, under super-synchronous mode of DFIG, the phase sequence becomes negative, and rotor three-phase currents are sequenced as a-c-b. In this case, The CADI is given as follows:

(34) $$CADI_{izn}=\left|-\theta_{zn}-\theta_{irn}\right|=\left|-\theta_{irnf}+\delta -\theta_{irn}\right|=\left|\delta \right|+\left|\theta_{irnf}+\theta_{irn}\right|$$

Under super-synchronous mode, for ITSC fault in rotor phase *a*, *b*, and *c*, the *CADI_izn_* values are given in Table 2, where CADI is the sum of IPO of FFC in ZSC signal and IPOs of the FFCs in three-phase current signals (*θ_zn_* + *θ_irn_*).

Under the sub-synchronous mode, for HRC fault, using (22) and (26), CADI is given as follows:

(35) $$CADI_{hzn}=\left|\theta_{zn}-\theta_{irn}-\pi \right|=\left|\theta_{irnf}+\pi -\theta_{irn}-\pi \right|=\left|\theta_{irnf}-\theta_{irn}\right|$$

where *CADI_hzn_* indicates CADI values under HRC fault. Using (26), (30) and (34), *CADI_hzn_* for HRC in phase-a is given as follows:

(36) $$\begin{array}{l} CADI_{hza}=\left|\pi -\Delta \delta_{hf}\right|+\left|\theta_{ira}-\pi -(\theta_{ira}+\Delta \theta_{ira})\right|\approx \left|-\Delta \delta_{hf}+\Delta \theta_{ira}\right|\\ CADI_{hzb}=\left|\pi -\Delta \delta_{hf}\right|+\left|\theta_{ira}-\pi -\theta_{irb}\right|=\left|\pi -\Delta \delta_{hf}\right|+\left|\theta_{ira}-\pi -(\theta_{ira}-2\pi /3-\Delta \theta_{ira})\right|\approx \left|\pi /3-\Delta \delta_{hf}+\Delta \theta_{ira}\right|\\ CADI_{hzc}=\left|\pi -\Delta \delta_{hf}\right|+\left|\theta_{ira}-\pi -\theta_{irc}\right|=\left|\pi -\Delta \delta_{hf}\right|+\left|\theta_{ira}-\pi -(\theta_{ira}+2\pi /3+\Delta \theta_{ira})\right|\approx \left|5\pi /3-\Delta \delta_{hf}+\Delta \theta_{ira}\right| \end{array}$$

So, for HRC in rotor phase-a, *CADI_hzn_* values are |−*∆δ_hf_* + *∆θ_ira_*|, |*π/3* − *∆δ_hf_* + *∆θ_ira_*|, and |5*π/3* − *∆δ_hf_* + *∆θ_ira_*| for rotor phases *a*, *b*, and *c*, respectively. Likewise, for HRC in rotor phases b and c, the values are given in Table 3. It can be seen from Table 3 that *CADI_hzn_* has the smallest value for the faulty rotor phase when ITSC fault occurs in one of the rotor three phases, where CADI is the difference between IPOs of FFC in ZSC signal and the three-phase current signals with π subtracted (*θ_zn_* − *θ_irn_* − π).

However, under the super-synchronous mode, for HRC, the CADI is given as follows:

(37) $$CADI_{hzn}=\left|-\theta_{zn}-\theta_{irn}-\pi \right|=\left|-\theta_{irnf}+\pi -\theta_{irn}-\pi \right|=\left|\theta_{irnf}+\theta_{irn}\right|$$

Under the super-synchronous mode, for HRC fault in rotor phases *a*, *b*, and, *c*, the *CADI_hzn_* values are given in Table 2, where CADI is the sum of IPO of FFC in ZSC signal and IPOs of the FFC in three-phase current signals (*θ_zn_* + *θ_irn_*).

### 3.5. Algorithm for DFIG Rotor Winding Faults Diagnosis

**Figure 4:**
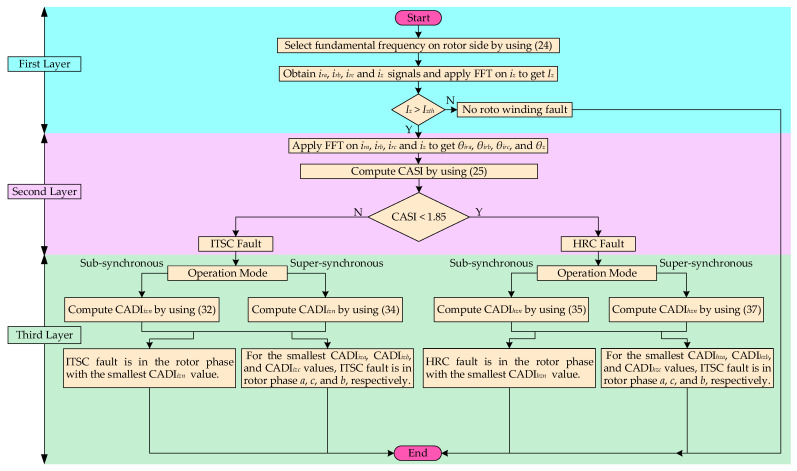
Algorithm based on three-layered ZSC-CASI-CADI framework for DFIG rotor winding faults diagnosis.

The algorithm for fault diagnosis is based on the three-layered *ZSC-CASI-CADI* framework and implements the proposed methodology for DFIG rotor winding faults detection, discrimination, and localization as shown in Figure 4. Details of the algorithm layers are given as follows:

First layer: The algorithm begins by measuring the three-phase rotor currents, ZSC signal and the slip. Based on the measured slip, the fundamental frequency of the rotor side is set according to (24). FFT is applied to separate the FFC magnitude (*I_z_*) from ZSC signal. The *I_z_* is compared with its threshold value *I_zth_* which is predetermined through the measurements under healthy operating conditions. If *I_z_* is more than *I_zth_*, it detects the occurrence of a fault in rotor winding. On the other hand, if *I_z_* is below *I_zth_*, it indicates that the rotor winding is healthy. The level to which the *I_z_* is more than *I_zth_* is a measure of the level of fault severity.

Second Layer: After the confirmation of the occurrence of a winding fault in the first layer, the type of fault is identified in the second layer. It discriminates between ITSC and HRC faults. FFT is applied to extract the IPOs of the FFCs from rotor three-phase current signals (*θ_ira_*, *θ_irb_*, and *θ_irc_*) and the ZSC signal (*θ*_z_). The fault type is discriminated by computing *CASI* values using (25). If the CASI value is greater than 1.85, the fault is discriminated as ITSC. On the other hand, if the CASI value is less than 1.85, the fault is discriminated as HRC.

Third Layer: After the confirmation of the type of winding fault in the second layer, the faulty phase is localized in the third layer. The operation mode of DFIG is recognized by the slip *s*. Sub-synchronous and super-synchronous modes are recognized by the positive and negative slip values, respectively. Faulty phase is localized by computing CADI values using (32), (34) for ITSC fault and (35), (37) for HRC fault, respectively. For ITSC fault, under the sub-synchronous operation mode, the faulty rotor phase has the smallest *CADI_izn_* value using (32). Meanwhile, under the super-synchronous mode, the faulty rotor phase is *a*, *b*, and *c* if it has the smallest *CADI_iza_*, *CADI_izc_*, and *CADI_izb_* value using (34), respectively. For HRC fault, under the sub-synchronous operation mode, the faulty rotor phase has the smallest *CADI_hzn_* value using (35). Meanwhile, under the super-synchronous mode, the faulty rotor phase is *a*, *b*, and *c* if it has the largest *CADI_hza_*, *CADI_hzc_*, and *CADI_hzb_* value using (37), respectively.

## 4. Simulations

To comprehensively validate the proposed three-layered ZSC- CASI- CADI diagnostic framework both for ITSC and HRC faults in DFIG rotor windings, an extensive simulation study has been conducted using MATLAB/Simulink (R2024a). The study involves both fault types with different severity levels under nearly realistic operating conditions. For ITSC faults, short circuits involving one, two, and five turns in rotor phase-a are simulated. For HRC faults, additional resistances of 0.2 mΩ, 0.5 mΩ, and 1 mΩ are connected in the same phase. These configurations for ITSC and HRC faults represent progressive severity levels and are useful for the evaluation of the methodology to detect the faults at their early stages when their severity levels are low. The simulations have been carried out considering different load and speed conditions, covering both sub-synchronous and super-synchronous modes of DFIG operation. The detailed parameters of the DFIG used in this simulation study are presented in Table 3.

The detectability of the rotor winding faults in DFIG is strongly influenced by the operating slip conditions. When the slip is high, the fault signatures are stronger and easier to detect. However, when the slip is low, these signatures become much weaker and make the fault diagnosis difficult. This is a key challenge because in real-world wind power applications, the DFIG is often required to operate with a very low slip to stay synchronized with the power grid efficiently [42,43]. Since this methodology has been designed for real-time applications, the simulations consider low-slip conditions where fault signatures are naturally weak. Therefore, to capture the variable speed and load conditions of a realistic DFIG, the simulations are carried out using a range of slip and load combinations that covers slip values from ±0.1% to ±10% and load levels from 50% to 100%.

### 4.1. Simulation Under DFIG Sub-Synchronous Operation Mode

Simulations under the sub-synchronous operation mode are conducted under variable operating conditions. These variable conditions are introduced by three distinct operation scenarios (OSs), each of which considers different load and speed combination for DFIG variable operation conditions.

- OS1: Operation of DFIG under load = 100%, and slip = 0.02
- OS2: Operation of DFIG under load = 70%, and slip = 0.04
- OS3: Operation of DFIG under load = 50%, and slip = 0.1

**Figure 5:**
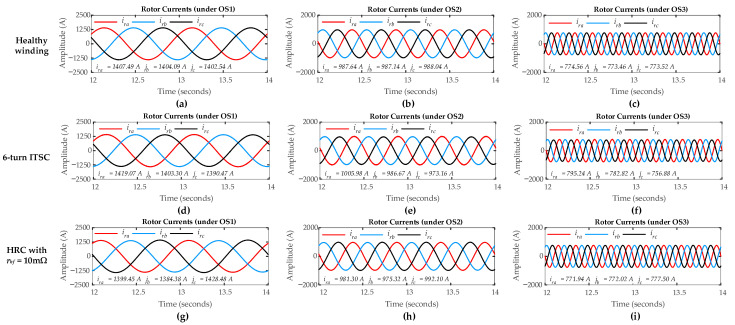
DFIG rotor three-phase currents: no fault condition under (**a**) OS1; (**b**) OS2; (**c**) OS3; six-turn ITSC condition in phase-a under (**d**) OS1; (**e**) OS2; (**f**) OS3; HRC with *r_hf_* = 10 mΩ in phase-a under (**g**) OS1; (**h**) OS2; (**i**) OS3.

The DFIG simulation is carried out in sub-synchronous mode with a positive slip value, where the healthy rotor winding is simulated considering no ITSC and HRC faults (*µ* = 0, *r_hf_* = 0 Ω). For ITSC fault, the rotor winding is simulated representing a six-turn short circuit in rotor phase-a with µ = 0.06. For HRC fault, the rotor winding is simulated by connecting a high resistance in phase-a with *r_hf_* = 0.01 Ω. Figure 5 illustrates the three-phase rotor currents for both healthy and faulty rotor windings under ITSC and HRC faults considering three different operating scenarios (OS1, OS2, and OS3). The rotor currents show a positive phase sequence (abc), confirming that the DFIG is operating in the sub-synchronous mode. Under healthy conditions (where *µ* = 0, *r_hf_* = 0 Ω), the current waveforms remain nearly symmetrical across all operating scenarios. However, in ITSC and HRC fault scenarios with *µ* = 0.06 for ITSC, and *r_hf_* = 0.01 Ω for HRC, the three-phase rotor currents show only minimal asymmetry, even under a high level of fault severity. As a result, the analysis of rotor current waveforms in the time domain proves insufficient for detecting and localizing the rotor winding faults, particularly in cases with low severity. To overcome this challenge, frequency components in the three-phase rotor currents and the ZSC signal are inspected using FFT to achieve more accurate fault detection.

The DFIG simulation has been conducted in sub-synchronous mode, analyzing both healthy and faulty rotor winding conditions. The healthy conditions include no ITSC and HRC faults (*µ* = 0, *r_hf_* = 0 Ω). The faulty conditions include an ITSC fault representing a six-turn short circuit in rotor phase-a (with *µ* = 0.06) and an HRC fault with an additional resistance (*r_hf_* = 0.001 Ω) in the same phase. Figure 5 shows the three-phase rotor currents under healthy, ITSC, and HRC fault conditions across three different operational scenarios (OS1, OS2, OS3). The positive phase sequence confirms the sub-synchronous mode operation.

For the healthy condition, the three-phase rotor currents remain nearly symmetrical. Under the ITSC and HRC faults, minor asymmetry is introduced. Critically, the time domain analysis of these rotor currents provides insufficient clarity for reliable detection and discrimination, especially for low-severity, incipient faults. To overcome this challenge, frequency components in the three-phase rotor currents and the ZSC signal are inspected using FFT to achieve more accurate fault detection.

**Figure 6:**
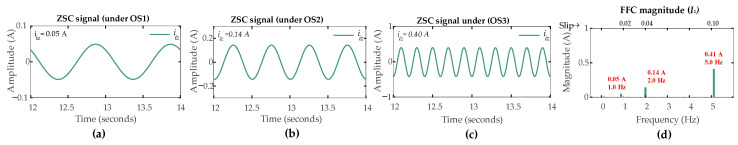
Results of DFIG simulation conducted under rotor winding healthy condition: ZSC under (**a**) OS1; (**b**) OS2; (**c**) OS3; (**d**) *Iz*.

Figure 6 shows the ZSC signals and their FFT analysis for the healthy condition under OS1, OS2, and OS3 operating scenarios.

The FFC observed in the ZSC signal directly corresponds to the rotor electrical frequency, which varies with varying operational slip across three different operating scenarios. As shown, this frequency measures 1 Hz, 2 Hz, and 5 Hz for operating scenarios OS1, OS2, and OS3, respectively, which is consistent with theoretical predictions derived from the slip–frequency relationship in (24).

Although in a perfectly symmetrical system there would, in principle, be a zero ZSC, there is always a small but fundamentally persistent component even in the case of healthy operation, owing to the imbalances inherent in DFIG and the measurement noise. The measured magnitudes of ZSC are [0.05 A, 0.14 A, 0.41 A] for the three scenarios [OS1, OS2, OS3], respectively. Therefore, a detection threshold *I_zth_* of 1 A is established in order to account for the ZSC magnitudes under healthy conditions. As per the first layer of the algorithm, any ZSC fundamental magnitude exceeding this threshold will trigger a fault alert and initiate the subsequent discrimination process.

**Figure 7:**
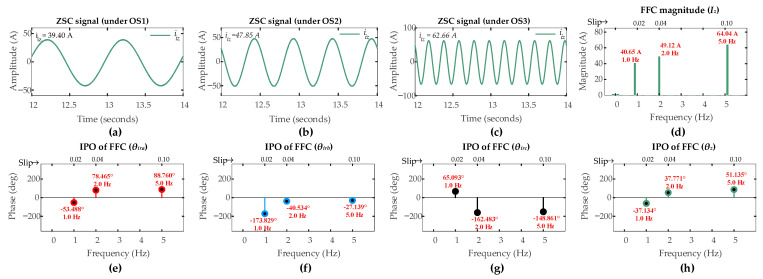
Results of DFIG simulation conducted under scenarios [OS1, OS2, OS3] for six-turn ITSC in phase-a of rotor winding: ZSC signal under (**a**) OS1; (**b**) OS2; (**c**) OS3; (**d**) *I_z_*; (**e**) *θ_ira_*; (**f**) *θ_irb_*; (**g**) *θ_irc_*; (**h**) *θ_z_*.

**Figure 8:**
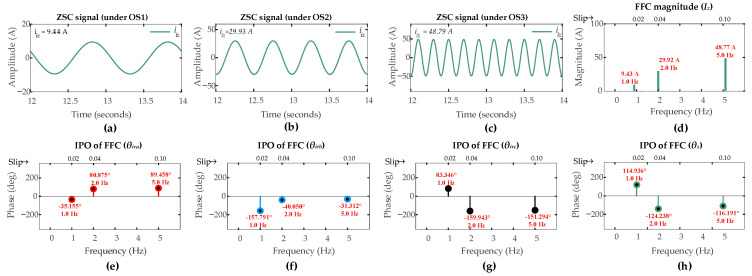
Results of DFIG simulation conducted under scenarios [OS1, OS2, OS3] for HRC with *r_hf_* = 10 mΩ in rotor winding phase-a: ZSC signal under (**a**) OS1; (**b**) OS2; (**c**) OS3; (**d**) *I_z_*; (**e**) *θ_ira_*; (**f**) *θ_irb_*; (**g**) *θ_irc_*; (**h**) *θ_z_*.

Figure 7 and Figure 8 present the ZSC signals and the corresponding FFT analysis for a six-turn ITSC fault (µ = 0.06) and an HRC fault (*r_hf_* = 10 mΩ) in rotor winding phase-a under three operating scenarios [OS1, SO2, OS3]. The significant increase in ZSC magnitudes relative to the healthy operation clearly reflects that both types of faults certainly introduce asymmetry in the rotor currents.

The FFT analysis reveals distinct fault signatures. For the six-turn ITSC fault in Figure 7, the FFC magnitude of ZSC signal measures [40.65 A, 49.12 A, 64.04 A] under operating scenarios [OS1, OS2, OS3], respectively.

For the HRC fault, it is shown in Figure 8 that the corresponding magnitudes are [9.43 A, 29.92 A, 48.77 A] under the respective operating scenarios. These values substantially exceed the pre-set threshold of 1 A across all operating conditions, successfully triggering the fault detection mechanism in first layer of the proposed algorithm for both fault types.

The second layer of the algorithm discriminates between two types of faults. The IPOs of the FFCs extracted from the ZSC signal and three-phase rotor currents provide the necessary data for fault discrimination and localization. For the ITSC fault, the IPOs in the FFC of the ZSC signals are −37.13°, 37.78°, and 51.14° under OS1, OS2, OS3, respectively. The IPOs in the FFCs of the rotor currents under the respective operating scenarios [S1, S2, S3] are [−53.50°, 78.51°, 88.80°], [−173.81°, −40.50°, −27.10°], and [65.11°, −162.50, −148.89°] for phases [*a*, *b*, *c*], respectively. For the HRC fault, the corresponding IPOs in the FFC of ZSC signals are 114.94°, −124.17°, and −116.20° under OS1, OS2, OS3, respectively. The IPOs in the FFCs of the rotor currents under the respective operating scenarios [S1, S2, S3] are [−35.16°, 80.88°, 89.46°], [−157.79°, −40.05, −31.32°], and [83.35°, −159.94, −151.30°] for phases [*a*, *b*, *c*], respectively.

The CASI values are computed using the phase angles in (25). The ITSC fault gives CASI values of [2.018, 2.023, 2.416], while the HRC fault gives CASI values of [1.538, 1.691, 1.652], under operating scenarios [OS1, OS2, OS3], respectively. CASI values are more than 1.85 for ITSC fault and less than 1.85 for HRC fault. Following the discrimination threshold of 1.85 from (31), the second layer of algorithm correctly discriminates between two types of faults across all operating scenarios.

The third layer of the algorithm localizes the fault. For ITSC fault under sub-synchronous mode, the CADI values [*CADI_iza_*, *CADI_izb_*, *CADI_izc_*] are computed using (32). CADI values for ITSC fault [*CADI_iza_*, *CADI_izb_*, *CADI_izc_*] are [16.37°, 136.67°, 102.23°], [40.7°, 78.30°, 200.30°], and [37.66°, 78.24°, 200.04°] under OS1, OS2, and OS3, respectively. The consistent smallest value of *CADI_iza_* under all operating scenarios accurately identifies phase-a as the fault location, which is in accordance with the third layer of algorithm. Similarly, for HRC fault, under the sub-synchronous mode, the CADI values [*CADI_hza_*, *CADI_hzb_*, *CADI_hzc_*] are computed using (35). CADI values for HRC fault [*CADI_hza_*, *CADI_hzb_*, *CADI_hzc_*] are [29.1°, 92.01°, 148.41°], [25.08°, 95.85°, 215.74°], and [25.66°, 95.12°, 215.09°] under OS1, OS2, and OS3, respectively. The consistent minimum value of *CADI_hza_* under all operating scenarios confirms the same faulty phase, indicating the consistent localization capability of the third layer of algorithm for different fault types.

Faults including six-turn ITSC and HRC with *r_hf_* = 10 mΩ are considered as high-severity faults. Therefore, to validate the sensitivity of the methodology to fault severity, the framework is tested with lower-severity ITSC and HRC faults under sub-synchronous operation. This analysis includes a three-turn ITSC (µ = 0.03) and a single-turn ITSC (µ = 0.01) in rotor phase-a for lower-severity ITSC faults. Correspondingly, lower-severity HRC faults are also analyzed with *r_h_* = 5 mΩ and *r_hf_* = 2 mΩ to provide a comprehensive assessment.

**Figure 9:**
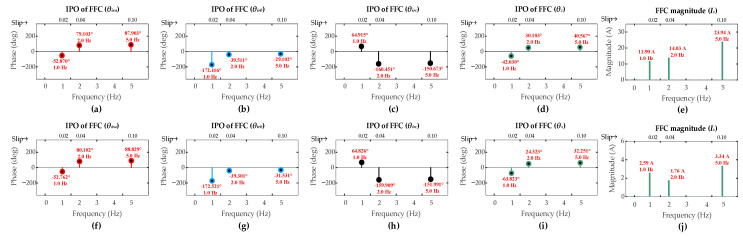
Results of DFIG simulation conducted under scenarios [OS1, OS2, OS3] for low-severity ITSCs in rotor winding phase-a: three-turn fault (**a**) *θ_ira_*; (**b**) *θ_irb_*; (**c**) *θ_irc_*; (**d**) *θ_z_*; (**e**) *I_z_*; one-turn fault (**f**) *θ_ira_*; (**g**) *θ_irb_*; (**h**) *θ_irc_*; (**i**) *θ_z_*; (**j**) *I_z_*.

**Figure 10:**
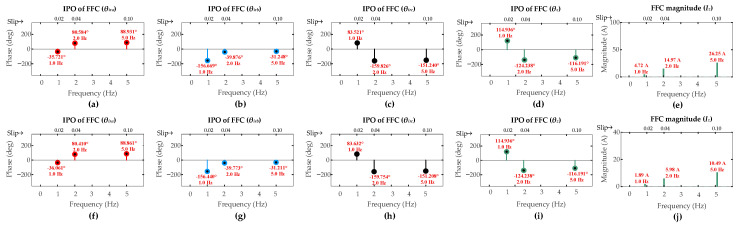
Results of DFIG simulation conducted under scenarios [OS1, OS2, OS3] for low-severity HRCs in rotor winding phase-a with *r_hf_* = 5 mΩ (**a**) *θ_ira_*; (**b**) *θ_irb_*; (**c**) *θ_irc_*; (**d**) *θ_z_*; (**e**) *I_z_*; with *r_hf_* = 2 mΩ; (**f**) *θ_ira_*; (**g**) *θ_irb_*; (**h**) *θ_irc_*; (**i**) *θ_z_*; (**j**) *I_z_*.

The FFT results for these low-severity faults are shown in Figure 9 and Figure 10. As expected, the FFC magnitude of the ZSC signal shows a direct correlation with fault severity levels. For the ITSC fault, these magnitudes for the three-turn and the single-turn cases are comparably lower than that of the six-turn fault across all operating scenarios [OS1, OS2, OS3]. A similar trend is observed for the HRC faults where the FFC magnitude of the ZSC signal decreases as *r_hf_* decreases.

The results for all fault severities under sub-synchronous mode are summarized in Table 4. It can be observed for both ITSC and HRC fault types that even the lowest severity cases produce an FFC magnitude of the ZSC that exceeds the pre-set detection threshold of 1 A. This successfully triggers the fault alarm, confirming the capability of the algorithm to detect low-severity faults in the first layer.

The CASI values for the low-severity faults are calculated. The three-turn ITSC fault gives CASI values of [2.268, 2.551, 2.586], while one-turn ITSC fault gives CASI values of [2.368, 2.813, 2.825], under operating scenarios [OS1, OS2, OS3], respectively. The HRC fault with *r_hf_* = 5 mΩ yields CASI values of [1.488, 1.617, 1.646], while the HRC fault with *r_hf_* = 2 mΩ yields CASI values of [1.474, 1.619, 1.646], under the respective operating scenarios. The CASI values remain consistently above the 1.85 threshold for low-severity ITSC faults and remain below this threshold for the low-severity HRC faults, ensuring the capability of the algorithm to accurately discriminate fault types with lower-severity levels in the second layer.

The CADI values for the low-severity faults are calculated. For three-turn ITSC fault, CADI values [*CADI_iza_*, *CADI_izb_*, *CADI_izc_*] are [10.84°, 130.38°, 106.95°], [49.51°, 69.60°, 190.60°], and [47.33°, 69.67°, 191.24°] under OS1, OS2, and OS3, respectively. For one-turn ITSC fault, the CADI values are [11.06°, 108.70°, 126.65°], [55.78°, 63.62°, 184.23°], and [56.58°, 62.78°, 184.23°] under the respective operating scenarios. For HRC fault with *r_hf_* = 5 mΩ, CADI values [*CADI_hza_*, *CADI_hzb_*, *CADI_hzc_*] are [29.34°, 91.61°, 148.58°], [24.75°, 95.71°, 144.35°], and [25.13°, 95.05°, 144.96°] under OS1, OS2, and OS3, respectively. For HRC fault with *r_hf_* = 2 mΩ, the CADI values are [29.12°, 91.38°, 148.69°], [24.58°, 95.60°, 144.42°], and [24.81°, 95.01°, 144.99°] under the respective operating scenarios. The consistent smallest values of *CADI_iza_* and *CADI_hza_* for ITSC and HRC faults, respectively, accurately identify phase-a as the fault location under all operating scenarios, confirming the capability of the algorithm to accurately identify the fault location under lower-severity levels in the third layer.

### 4.2. Simulation Under DFIG Super-Synchronous Operation Mode

Simulations under super-synchronous operation mode are conducted under variable operating conditions. These variable conditions are introduced by three distinct operation scenarios (OSs), each of which considers different load and speed combination for DFIG variable operation conditions.

- OS4: Operation of DFIG under load = 60%, and slip = −0.03
- OS5: Operation of DFIG under load = 75%, and slip = −0.06
- OS6: Operation of DFIG under load = 100%, and slip = −0.08

**Figure 11:**
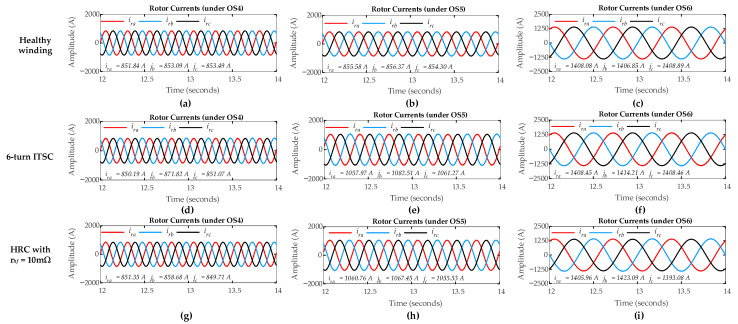
DFIG rotor three-phase currents: no fault condition under (**a**) OS4; (**b**) OS5; (**c**) OS6; six-turn ITSC condition in phase-a under (**d**) OS4; (**e**) OS5; (**f**) OS6; HRC with *r_hf_* = 10 mΩ in phase-a under (**g**) OS4; (**h**) OS5; (**i**) OS6.

To further validate the methodology under super-synchronous operation, DFIG simulation is carried out with negative slip values. The simulation considers a healthy rotor winding with no faults (*µ* = 0, *r_hf_* = 0 Ω) and a faulty rotor winding with a six-turn ITSC fault in phase-a (µ = 0.06), and a corresponding HRC fault in the same phase (*r_hf_* = 10 mΩ). Figure 11 shows the three-phase rotor currents for healthy, ITSC, and HRC fault conditions under three super-synchronous operating scenarios (OS4, OS5, OS6). The negative phase sequence (acb-phase sequence) confirms that the DFIG operation mode is super-synchronous. For the healthy condition, the rotor currents remain nearly symmetrical. Under both the ITSC and HRC fault conditions, the three-phase currents show only minimal asymmetry which is not prominent enough for reliable visual diagnosis in the time domain, even for these relatively high-severity faults. Consequently, as conducted in the sub-synchronous mode case, the analysis of fundamental frequency components in the three-phase rotor currents and the ZSC signal via FFT is essential for effective fault diagnosis in super-synchronous operation which forms the basis for the proposed three-layer diagnostic framework.

**Figure 12:**
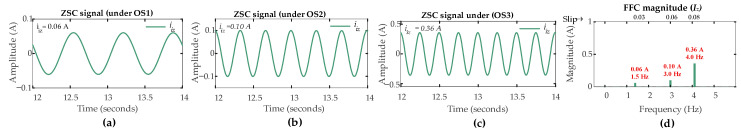
Results of DFIG simulation conducted under rotor winding healthy condition: ZSC under (**a**) OS4; (**b**) OS5; (**c**) OS5; (**d**) *Iz*.

Figure 12 displays the ZSC waveforms and their corresponding FFT analyses for the healthy rotor winding (µ = 0, *r_hf_* = 0 Ω) under operating scenarios OS4, OS5, and OS6. The rotor fundamental frequency in the ZSC varies with the operational slip. FFT measures FFC in ZSC 1.5 Hz, 3 Hz, and 4 Hz for OS4, OS5, and OS6, respectively. These values align precisely with the theoretical relationship given by (24).

A perfectly balanced system would yield negligible ZSC, but a small ZSC is present due to inherent machine imperfections.

The measured FFC magnitudes of the ZSC for the healthy winding are 0.06 A, 0.10 A, and 0.36 A under OS4, OS5, and OS6, respectively. Hence, the detection threshold *I_zth_* is maintained at 1 A in super-synchronous mode also. As defined in the first layer of the proposed algorithm, any FFC magnitude of the ZSC exceeding this pre-set threshold will indicate the presence of either an ITSC or an HRC fault in the rotor winding.

**Figure 13:**
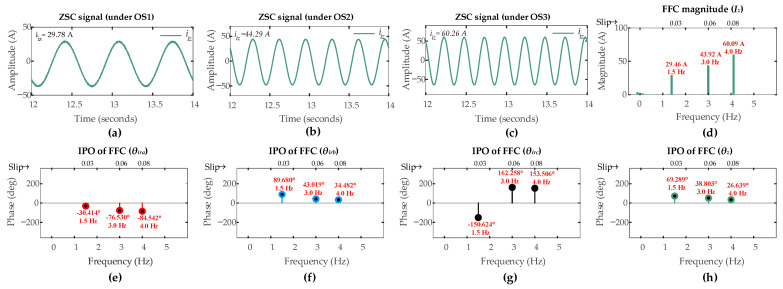
Results of DFIG simulation conducted under scenarios [OS4, OS5, OS6] for six-turn ITSC in rotor winding phase-a of rotor winding: ZSC signal under (**a**) OS4; (**b**) OS5; (**c**) OS6; (**d**) *I_z_*; (**e**) *θ_ira_*; (**f**) *θ_irb_*; (**g**) *θ_irc_*; (**h**) *θ_z_*.

**Figure 14:**
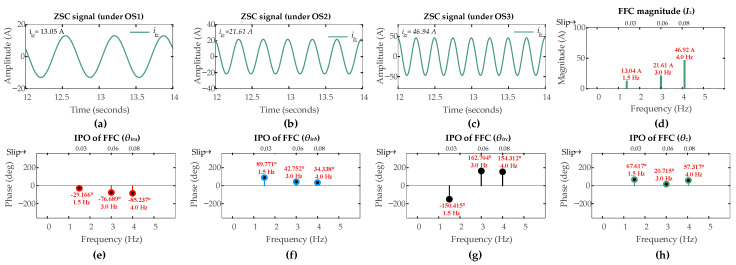
Results of DFIG simulation conducted under scenarios [OS4, OS5, OS6] for HRC with *r_hf_* = 10 mΩ in rotor winding phase-a: ZSC signal under (**a**) OS4; (**b**) OS5; (**c**) OS6; (**d**) *I_z_*; (**e**) *θ_ira_*; (**f**) *θ_irb_*; (**g**) *θ_irc_*; (**h**) *θ_z_*.

Figure 13 and Figure 14 present the ZSC signals and corresponding FFT analysis for a six-turn ITSC fault (µ = 0.06) and an HRC fault (*r_hf_* = 10 mΩ) in rotor phase-a under super-synchronous operating scenarios [OS4, OS5, OS6]. The significant increase in ZSC magnitude relative to healthy operation confirms the noticeable system asymmetry introduced by both types of faults.

The FFT analysis reveals different fault signatures. For the six-turn ITSC fault in Figure 13, the FFC magnitudes of the ZSC measures are [29.77 A, 44.28 A, 60.24 A] under OS4, OS5, and OS6, respectively. For the HRC fault in Figure 14, the corresponding magnitudes are [13.04 A, 21.61 A, 46.92 A]. These values noticeably exceed the pre-set threshold of 1 A and successfully trigger the fault detection mechanism in the first layer of the algorithm for both fault types across all super-synchronous operating conditions.

The second layer of the algorithm proceeds with fault discrimination. The IPOs of the FFCs extracted from the ZSC and three-phase rotor currents provide the required data. For the ITSC fault, the IPOs in the FFC of ZSC signals are 69.29°, 38.80°, and 26.68° under OS4, OS5, OS6, respectively. The IPOs in rotor currents FFCs under the respective operating scenarios [SO4, SO5, SO6] are [−30.41°, −76.53°, −84.54°], [89.68°, 43.02°, 34.48°], and [−150.61°, 162.26, 153.51°] for phases [*a*, *b*, *c*], respectively. For the HRC fault, the corresponding IPOs in the FFC of ZSC signals are 67.62°, 20.72°, and 57.32° under OS4, OS5, OS6, respectively. The IPOs in rotor currents FFCs under the respective operating scenarios [SO4, SO5, SO6] are [−29.17°, −76.70°, −85.24°], [89.77°, 42.75, 34.34°], and [−150.42°, 162.71, 154.31°] for phases [*a*, *b*, *c*], respectively.

The CASI values are computed using the phase angles in (25). The ITSC fault gives CASI values of [1.886, 2.708, 2.511], while the HRC fault gives CASI values of [1.724, 1.767, 1.746], under operating scenarios [SO4, SO5, SO6], respectively. Following the discrimination threshold of 1.85 from (31), the second layer correctly discriminates between the two fault types across all super-synchronous operation scenarios.

The third layer of the algorithm localizes the fault. For ITSC fault, under super-synchronous mode, the CADI values [*CADI_iza_*, *CADI_izb_*, *CADI_izc_*] are computed using (34). These values are [38.80°, 158.97°, 81.32°], [37.73°, 81.82°, 201.06°], and [57.86°, 61.16°, 180.19°] under OS4, OS5, and OS6, respectively. The *CADI_iza_* being consistently small under all operating scenarios accurately identifies phase-a as the fault location, which is in accordance with the third layer of algorithm for super-synchronous mode. Similarly, for HRC fault, under super-synchronous mode, the CADI values [*CADI_hza_*, *CADI_hzb_*, *CADI_hzc_*] are computed using (37). These values for HRC fault are [38.45°, 157.39°, 82.80°], [55.98°, 63.47°, 183.43°], and [27.92°, 91.66°, 211.63°] under SO4, SO5, and SO6, respectively. The consistent minimum value of *CADI_hza_* under all operating scenarios confirms the same fault location, demonstrating the consistent localization capability of the third layer of algorithm for different fault types in super-synchronous mode.

To further validate the sensitivity of methodology under super-synchronous operation, the framework is tested with lower-severity faults. The analysis is extended to include a three-turn ITSC fault (µ = 0.03) and a single-turn ITSC fault (µ = 0.01) in rotor phase-a. Correspondingly, lower-severity HRC faults with *r_hf_* = 5 mΩ and *r_hf_* = 2 mΩ are also analyzed to provide a comprehensive assessment across varying fault severities.

**Figure 15:**
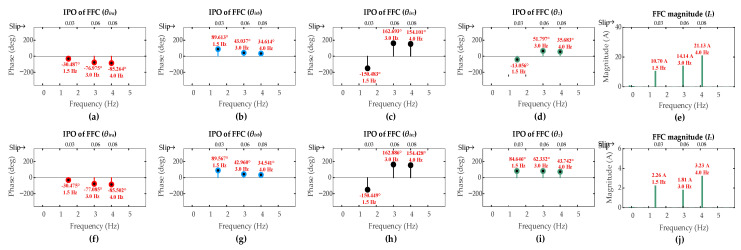
Results of DFIG simulation conducted under scenarios [OS4, OS5, OS6] for low-severity ITSCs in rotor winding phase-a: three-turn fault (**a**) *θ_ira_*; (**b**) *θ_irb_*; (**c**) *θ_irc_*; (**d**) *θ_z_*; (**e**) *I_z_*; one-turn fault (**f**) *θ_ira_*; (**g**) *θ_irb_*; (**h**) *θ_irc_*; (**i**) *θ_z_*; (**j**) *I_z_*.

**Figure 16:**
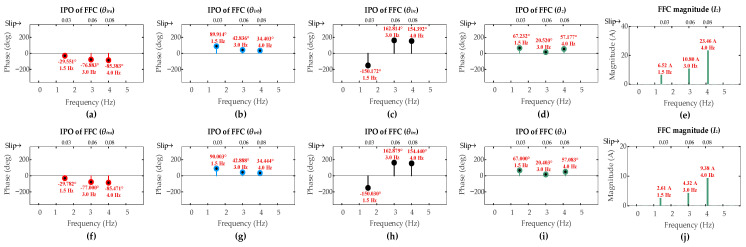
Results of DFIG simulation conducted under scenarios [OS4, OS5, OS6] for low-severity HRCs in rotor winding phase-a with *r_hf_* = 5 mΩ (**a**) *θ_ira_*; (**b**) *θ_irb_*; (**c**) *θ_irc_*; (**d**) *θ_z_*; (**e**) *I_z_*; with *r_hf_* = 2 mΩ (**f**) *θ_ira_*; (**g**) *θ_irb_*; (**h**) *θ_irc_*; (**i**) *θ_z_*; (**j**) *I_z_*.

Figure 15 and Figure 16 show the FFT results for low-severity faults under super-synchronous scenarios [ SO4, SO5, SO6]. As anticipated, the FFC magnitude of the ZSC signal shows a direct correlation with fault severity levels.

For the ITSC fault, these magnitudes for the three-turn and the single-turn cases are comparably lower than that of the six-turn fault across all operating scenarios. A similar trend is observed for the HRC faults where the FFC magnitude of the ZSC signal decreases as *r_hf_* decreases.

The results for all fault severities under super-synchronous mode are summarized in Table 5. It can be observed for both ITSC and HRC fault types that even the lowest severity cases produce an FFC magnitude of the ZSC that exceeds the pre-set detection threshold of 1 A. This successfully triggers the faults alarm, confirming the capability of the algorithm to detect low-severity faults in the first layer.

The CASI values for the low-severity faults are calculated. The three-turn ITSC fault gives CASI values of [2.063, 2.495, 2.691], while one-turn ITSC fault gives CASI values of [2.725, 1.928, 2.492], under operating scenarios [OS4, OS5, OS6], respectively. The HRC fault with *r_hf_* = 5 mΩ yields CASI values of [1.488, 1.617, 1.646], while the HRC fault with *r_hf_* = 2 mΩ yields CASI values of [1.726, 1.772, 1.756], under operating scenarios [SO4, SO5, SO6], respectively. The CASI values remain consistently above the 1.85 threshold for low-severity ITSC faults and remain below this threshold for the low-severity HRC faults, ensuring the capability of algorithm to accurately discriminate fault types under lower-severity levels in the second layer.

The CADI values for the low-severity faults are calculated. For three-turn ITSC faults, CADI values [*CADI_iza_*, *CADI_izb_*, *CADI_izc_*] are [43.53°, 76.55°, 163.54°], [24.73°, 94.82°, 214.06°], and [48.86°, 70.16°, 189.19°] under OS4, OS5, and OS6, respectively. For one-turn ITSC faults, the CADI values are [54.24°, 174.33°, 56.96°], [14.68°, 105.34°, 224.93°], and [41.52°, 78.35°, 197.84°] under the respective operating scenarios. For HRC fault with *r_hf_* = 5 mΩ, CADI values [*CADI_hza_*, *CADI_hzb_*, *CADI_hzc_*] are [37.68°, 157.14°, 82.94°], [56.36°, 63.36°, 183.33°], and [28.29°, 91.58°, 211.57°] under SO4, SO5, and SO6, respectively. For HRC fault with *r_hf_* = 2 mΩ, the CADI values are [37.22°, 157.00°, 83.03°], [56.60°, 63.29°, 183.28°], and [28.39°, 91.53°, 211.52°] under the respective operating scenarios.

The consistent smallest values of *CADI_iza_* and *CADI_hza_* for ITSC and HRC faults, respectively, accurately identify phase-a as the fault location under all operating scenarios, confirming the capability of algorithm to also accurately identify the fault location under lower-severity levels in the third layer under the super-synchronous mode.

### 4.3. Comparison with Existing Techniques

Table 6 gives the computational burden and latency comparisons of the proposed ZSC-CASI-CADI framework with the existing techniques. The proposed framework only requires one FFT calculation for each signal window. After the FFT, it simply requires a few basic calculations, such as ratios and phase differences, to obtain the fault indicators. Therefore, it does not require much time or memory which makes it fast and lightweight. Even with a large amount of data, it keeps the processing time low. The zero-sequence feature method also uses FFT but adds more steps, such as filtering and processing to isolate the imbalances in the system. While the calculations are not that complex, they still take more time compared to the proposed framework due to the extra filtering and calculations [44]. Park’s vector (modulus/angle) method involves abc to αβ/dq transformation and then calculating the magnitude and angle. Although these are simple calculations, the method still requires extra steps beyond the basic FFT, and more time is spent on this transformation and filtering, making it slower than the proposed method [45]. MCSA sideband method is the most computationally demanding because it requires multiple FFT calculations for different frequencies to detect faults. Each additional FFT increases the computational load. So, as the system becomes more complex, such as being under lower slip or during faults, this method requires more time and memory compared to the proposed method [46].

The proposed framework simplifies the computation by focusing on a single FFT, which is then followed by calculating the CASI and CADI using straightforward mathematical formulas based on the IPOs of the FFCs in the signals while other methods require additional filtering, transformations, and multiple FFT windows. As a result, the proposed framework has light computational burden and very low latency.

### 4.4. Detection Accuracy

Table 7 gives the detection accuracy comparison of the proposed ZSC-CASI-CADI framework with the existing techniques. The proposed framework is benchmarked against three widely used fault detection techniques: Negative-sequence features, Park’s vector (modulus/angle), and MCSA sidebands at the grid and rotor frequency. All methods are evaluated under the same operational scenarios (OS1–OS6, 1–2% harmonic content and both grid side and rotor sides filters enabled, using Intel i7 2.3 GH, 32 GB RAM). The accuracy for each method is measured under the same conditions, and the results are summarized in Table 7. While the accuracy is less in other methods due the factors described in Section 4.3, the proposed framework achieves an accuracy between 97% and 99%.

## 5. Discussion

The simulation results across all operating scenarios [OS1–OS6] and varying fault severity levels demonstrate the consistent and accurate performance of the proposed three-layer ZSC-CASI-CADI framework. The methodology successfully detects both ITSC and HRC faults in the first layer, correctly discriminates between them in the second layer and accurately identifies the faulty phase in the third layer. This validation confirms the framework can be effectively deployed for online rotor winding fault diagnosis in DFIG under variable load and speed conditions. An important advantage of the proposed framework is its computational efficiency because it relies on fundamental frequency component analysis rather than complex signal processing and thereby saves time and computational resources.

It is important to note that the rotor currents have 1–2% harmonics in the current study. The rotor side control loops influence system dynamics, particularly the rotor currents and their phase angles, which may affect the indicators during transient conditions or faults. However, the indicators remain robust because the framework focus on the FFC by using FFT. The grid disturbances, such as voltage sags or swells, can introduce noise, which may impact ZSC due to phase voltage imbalances. Yet, the FFT-based extraction of the framework proves to be robust against moderate noise and fluctuations. Also, the simulation system incorporates both grid side and rotor side filters that play a key role in reducing the impact of converter non-idealities by suppressing the higher-order harmonics.

The framework uses an adaptive FFT window length that varies depending on the rotor side frequency, *f_r_*, given in (24). The window length is calculated to capture five full cycles of the rotor side frequency every time to ensure sufficient frequency resolution for accurate fault detection. The window length is given as window length (seconds)= 5/*f_r_*. As the DFIG slip varies, the rotor frequency changes and the window length adapts accordingly. By dynamically adjusting the FFT window length in real time based on the rotor frequency, the framework maintains optimal frequency resolution while minimizing the computational delays and ensuring the applicability in real-time fault detection under varying operating conditions.

While the simulation results give strong confidence in the core idea behind the proposed method, turning it into a practical, real-time monitoring system introduces several real-world challenges. In a real-time operating environment, the quality of the measured signals can be easily affected by electromagnetic interference and natural noise in the sensors. These disturbances can hide the small fault features, especially during the early stages of the winding faults, which may either lead to unnecessary false alarms or wrong detections. In addition, the small manufacturing tolerances in the machine itself and the minor inaccuracies or imbalances in the measurement hardware can also create small baseline offsets. If these are not properly accounted for, they can appear similar to fault signatures. This means the system must include a reliable method for setting and adjusting the detection thresholds. Another concern is the delay caused by the real-time computation. Algorithms such as FFT or phasor extraction require processing time, and any latency may slow down the response of the system between the moment a fault begins and the moment it is detected.

For the framework to function reliably for DFIG winding fault diagnosis in real time, future developments should focus on better signal conditioning and noise reduction strategies, adaptive thresholds that automatically adjust to operating conditions and a faster and optimized hardware to keep the detection delays as low as possible.

## 6. Conclusions

This paper introduces a complete three-layered diagnostic framework, ZSC-CASI-CADI, for the detection, discrimination and localization of rotor winding faults in DFIG operating under varying conditions. By examining the DFIG mathematical model, the study identifies clear electrical signatures for both ITSC and HRC winding faults. The first layer of the framework uses the FFC of the ZSC signal to detect the presence of a fault. The second layer then uses the relationship of the IPOs between the ZSC signal and the rotor three-phase current signals, and quantifies through CASI to distinguish between ITSC and HRC faults. Finally, the third layer uses the CADI to locate the faulty phase in the rotor windings.

To implement the framework, an efficient and reliable algorithm is developed. The DFIG model covers a range of fault severities, including ITSC faults with one, three, and six ITSCs, and HRC faults with added resistances of 2 mΩ, 5 mΩ, and 10 mΩ. A wide set of simulations is carried out in MATLAB/Simulink (R2024a), covering six different operating scenarios [OS1–OS6] under both sub-synchronous and super-synchronous modes. The results show that the proposed method can detect low-severity faults, correctly discriminate between ITSC and HRC fault types, and accurately locate the faulty rotor phase in DFIG rotor windings.

The main contributions and findings of this study are summarized as follows:

(1) This study fills an important gap in the DFIG rotor winding fault diagnosis by explicitly validating the proposed framework under super-synchronous operation and dynamic low-frequency rotor conditions, which are often ignored in the existing literature. The results show that the ZSC-CASI-CADI framework performs consistently even when the rotor operates under challenging dynamic conditions across both sub-synchronous and super-synchronous modes. This strong performance across the full operating range of the DFIG greatly improves the practical value of the method for real-world wind energy application.
(2) The magnitude of FFC in the ZSC signal proves to be a very sensitive indicator for detecting rotor winding faults in DFIG, especially when the faults are still in the early, low-severity stage. However, obtaining the ZSC signal requires modification in the ∆-connected rotor windings for placing extra measurement hardware, which adds complexity to the overall rotor winding design. Because of this, the method is especially well-suited for large high-cost DFIG installations where the improved diagnostic accuracy compensates for the added hardware requirements.
(3) The IPO of FFC in the ZSC signal shows different sensitivity characteristics for different rotor winding fault types. For ITSC faults, this angle is highly sensitive to both the fault severity level and the DFIG operating conditions. For HRC faults, this angle remains largely consistent and shows only negligible variation with changes in fault severity or DFIG operating conditions. Meanwhile, the IPOs of the FFCs in the rotor three-phase currents show only minor sensitivity to both ITSC and HRC faults. This fundamental difference in the behavior of IPO of the FFC in the ZSC signal forms the theoretical basis for the fault discrimination (CASI) and localization (CADI) indicators, enabling reliable fault-type discrimination and accurate faulty phase localization
(4) The logic for fault localization depends on both the DFIG operating mode and the type of rotor winding fault. In sub-synchronous operation, where the rotor currents follow a positive (abc) phase sequence, the smallest angular difference identifies the faulty phase. For an ITSC fault, this difference is computed using (32) as the difference between the IPO of the FFC in the ZSC and the IPO of the FFC in each rotor phase current. The smallest value among *CADI_iza_*, *CADI_izb_*, *CADI_izc_* therefore corresponds to a fault in phase *a*, *b*, or *c*, respectively. For an HRC fault, the localization uses Equation (35), which introduces a π-shift in the calculation, but the principle remains the same and the corresponding phase with the smallest CADI value is identified as the faulty one. In super-synchronous operation, where the rotor currents follow a negative (acb) phase sequence, the localization logic adjusts accordingly, and the smallest angular summation identifies the faulty phase. For an ITSC fault, this summation is computed using (34) as the sum of the IPO of the FFC in the ZSC and the IPO of the FCC in each rotor phase current. The minimum among *CADI_iza_*, *CADI_izb_*, and *CADI_izc_* now corresponds to faults in phases *a*, *c*, and *b*, respectively. For an HRC fault, this specific mapping for localization is similarly applied using (37) and the corresponding phase with the smallest CADI value is identified as the faulty one. Together, these rules ensure that the CADI provides accurate and consistent phase identification for both fault types across all DFIG operating modes.

Future work would include hardware-in-the-loop validation of the proposed ZSC-CASI-CADI framework using a dedicated DFIG test rig. Building a laboratory-scale setup would allow controlled emulation of the real-time rotor winding fault scenarios, including both ITSC and HRC faults with different severity levels. Such a platform would provide the means to assess the real-time performance of the proposed framework under practical operating conditions. It would help to evaluate the impact of measurement noise and verify the robustness of the diagnostic indicators for DFIG health monitoring in a real-time environment.

## Figures and Tables

**Table 1:** *∆_izn_* values for rotor winding ITSC and HRC faults, incorporating the effects of faults and DFIG operating conditions.

Fault	Faulty Phase	*∆_izn_* = {*∆_iza_*, *∆_izb_*, *∆_izc_*}	Modified *∆_izn_* = {*∆_iza_*, *∆_izb_*, *∆_izc_*}
ITSC	Phase-a	{−*π + δ*, −π/3 − *δ*, π/3 + *δ*}	{(−*π* − *∆θ_ira_*) + (*δ* − *∆δ_if_*), (−π/3 + *∆θ_ira_*) + (*δ* − *∆δ_if_*), (π/3 − *∆θ_ira_*) + (*δ* − *∆δ_if_*)}
	Phase-b	{π/3 + *δ*, −*π + δ*, −π/3 − *δ*}	{(π/3 − *∆θ_ira_*) + (*δ* − *∆δ_if_*), (−*π* − *∆θ_ira_*) + (*δ* − *∆δ_if_*), (−π/3 + *∆θ_ira_*) + (*δ* − *∆δ_if_*)}
	Phase-c	{−π/3 − *δ*, π/3 + *δ*, −*π + δ*}	{(−π/3 + *∆θ_ira_*) + (*δ* − *∆δ_if_*), (π/3 − *∆θ_ira_*) + (*δ* − *∆δ_if_*), (−*π* − *∆θ_ira_*) + (*δ* − *∆δ_if_*)}
HRC	Phase-a	{0, 2π/3, −2π/3}	{(−*∆θ_ira_* − *∆δ_hf_*), (2π/3 + *∆θ_ira_* − *∆δ_hf_*), (−2π/3 − *∆θ_ira_* − *∆δ_if_*)}
	Phase-b	{−2π/3, 0, 2π/3}	{(−2π/3 − *∆θ_ira_* − *∆δ_if_*), (-*∆θ_ira_* − *∆δ_hf_*), (2π/3 + *∆θ_ira_* −*∆δ_hf_*)}
	Phase-c	{2π/3, −2π/3, 0}	{(2π/3 + *∆θ_ira_* − *∆δ_hf_*), (−2π/3 − *∆θ_ira_* − *∆δ_if_*), (−*∆θ_ira_* − *∆δ_hf_*)}

**Table 2:** Location indicator for rotor winding ITSC and HRC faults under two operation modes.

Fault and Mode	Faulty Phase	CADI Values	Location Indicator
ITSC (Sub-Synchronous) Using (32)	Phase-a	{(*δ* − *∆δ_if_* + *∆θ_ira_*, *δ* − *∆δ_if_* + *∆θ_ira_* +2π/3, *δ* − *∆δ_if_* + *∆θ_ira_* + 2π/3}	Smallest *CADI_iza_*
	Phase-b	{(*δ* − *∆δ_if_* + *∆θ_ira_* + 2π/3, *δ* − *∆δ_if_* + *∆θ_ira_*, *δ* − *∆δ_if_* + *∆θ_ira_* + 2π/3}	Smallest *CADI_izb_*
	Phase-c	{(*δ* − *∆δ_if_* +*∆θ_ira_* + 2π/3, *δ* − *∆δ_if_* + *∆θ_ira_* + 2π/3, *δ* − *∆δ_if_* + *∆θ_ira_*}	Smallest *CADI_izc_*
ITSC (Sup-Synchronous) Using (34)	Phase-a	{(*δ* − *∆δ_if_* +*∆θ_ira_*, *δ* − *∆δ_if_* +*∆θ_ira_* + 2π/3, *δ* − *∆δ_if_* + *∆θ_ira_* + 2π/3}	Smallest *CADI_iza_*
	Phase-b	{(*δ* − *∆δ_if_* + *∆θ_ira_* +2π/3, *δ* − *∆δ_if_* +*∆θ_ira_* + 2π/3, *δ* − *∆δ_if_* + *∆θ_ira_*}	Smallest *CADI_izc_*
	Phase-c	{(*δ* − *∆δ_if_* + *∆θ_ira_* + 2π/3, *δ* − *∆δ_if_* + *∆θ_ira_*, *δ* − *∆δ_if_* +*∆θ_ira_* + 2π/3}	Smallest *CADI_izb_*
HRC (Sub-Synchronous) Using (35)	Phase-a	{π − *∆δ_hf_* + *∆θ_ira_*, π/3 − *∆δ_hf_* + *∆θ_ira_*, π/3 − *∆δ_hf_* + *∆θ_ira_*}	Smallest *CADI_hza_*
	Phase-b	{π/3 − *∆δ_hf_* + *∆θ_ira_*, π − *∆δ_hf_* + *∆θ_ira_*, π/3 − *∆δ_hf_* +*∆θ_ira_*}	Smallest *CADI_hzb_*
	Phase-c	{π/3 − *∆δ_hf_* +*∆θ_ira_*, π/3 − *∆δ_hf_* +*∆θ_ira_*, π − *∆δ_hf_* + *∆θ_ira_*}	Smallest *CADI_hzc_*
HRC (Sup-Synchronous) Using (37)	Phase-a	{−*∆δ_hf_* + *∆θ_ira_*, 5π/3 − *∆δ_hf_* + *∆θ_ira_*, π/3 − *∆δ_hf_* + *∆θ_ira_*}	Smallest *CADI_hza_*
	Phase-b	{5π/3 − *∆δ_hf_* + *∆θ_ira_*, π/3 − *∆δ_hf_* +*∆θ_ira_*, *-∆δ_hf_* + *∆θ_ira_*}	Smallest *CADI_hzc_*
	Phase-c	{π/3 − *∆δ_hf_* + *∆θ_ira_*, −*∆δ_hf_* + *∆θ_ira_*, 5π/3 − *∆δ_hf_* + *∆θ_ira_*}	Smallest *CADI_hzb_*

**Table 3:** Simulation parameters of the DFIG.

Property	Value	Property	Value
DFIG rated power	2 MW	Number of poles	4
RMS rotor voltage (L-L)	207 V	RMS stator voltage (L-L)	690 V
Rotor current (RMS)	1000 A	Stator current (RMS)	1760 A
Per-phase rotor resistance	0.00292 Ω	Per-phase stator resistance	0.00261 Ω
Rotor leakage inductance	783 µH	Stator leakage inductance	87 µH
Magnetizing inductance	2.5 mH	Windings (stator/rotor)	Star/delta
Rotor per-phase turns	100	Grid side frequency	50 Hz
Stator per-phase turns	333	Synchronous speed	1500 r/min

**Table 4:** Results of simulation conducted under sub-synchronous operating scenarios [OS1, OS2, OS3].

Fault Level	OS Case	Mag. and IPO of FFC in ZSC {*I_z (A)_, θ_z (deg_._)_*}	IPOs of FFCs in Rotor Currents {*θ_ira_, θ_irb_, θ_irc(deg_._)_*}	CASI Value	CADI Values {CASI*_za_*, CASI*_zb_*, CASI*_zc_*}	Diagnostics
0	OS1	{0.05, N/A}	*I_z_* < *I_zth_*			No rotor winding fault detected
	OS2	{0.14, N/A}				
	OS3	{0.41, N/A}				
ITSC *µ* = 0.06	OS1	{40.65, −73.82°}	{−53.50°, −173.81°, 65.11°}	1.912	{16.37°, 136.67°, 102.23°}	CASI > 1.85, *CADI_iza_* is smallest_,_ ITSC in phase-a
	OS2	{49.12, 37.78°}	{78.51°, −40.50°, −162.50°}	2.023	{40.7°, 78.30°, 200.30°}	
	OS3	{64.04, 45.30°}	{88.80°, −27.10°, −148.89°}	2.146	{37.66°, 78.24°, 200.04°}	
ITSC *µ* = 0.03	OS1	{11.90, −42.03°}	{−52.870°, −172.42°, 64.92°}	2.268	{10.84°, 130.38°, 106.95°}	CASI > 1.85, *CADI_iza_* is smallest_,_ ITSC in phase-a
	OS2	{14.3, 30.10°}	{79.61°, −39.50°, −160.50°}	2.551	{49.51°, 69.60°, 190.60°}	
	OS3	{23.94, 40.57°}	{87.90°, −29.10°, −150.67°}	2.586	{47.33°, 69.67°, 191.24°}	
ITSC *µ* = 0.01	OS1	{2.59, −63.82°}	{−52.76°, −172.52°, 64.83°}	2.368	{11.06°, 108.70°, 126.65°}	CASI > 1.85, *CADI_iza_* is smallest_,_ ITSC in phase-a
	OS2	{1.76, 24.32°}	{80.10°, −39.30°, −159.91°}	2.813	{55.78°, 63.62°, 184.23°}	
	OS3	{3.34, 32.25°}	{88.83°, −30.53°, −151.99°}	2.825	{56.58°, 62.78°, 184.23°}	
HRC *r_if_* = 10 mΩ	OS1	{9.43, 114.94°}	{−35.16°, −157.79°, 83.35°}	1.538	{29.1°, 92.01°, 148.41°}	CASI < 1.85, *CADI_hza_* is smallest_,_ HRC in phase-a
	OS2	{29.92, −124.17°}	{80.88°, −40.50°, −159.94°}	1.691	{25.08°, 95.85°, 215.74°}	
	OS3	{48.77, −116.20°}	{89.46°, −31.32°, −151.30°}	1.652	{25.66°, 95.12°, 215.09°}	
HRC *r_if_* = 5 mΩ	OS1	{4.72, 114.94°}	{−35.72°, −156.67°, 83.52°}	1.488	{29.34°, 91.61°, 148.58°}	CASI < 1.85, *CADI_hza_* is smallest_,_ HRC in phase-a
	OS2	{14.97, −124.17°}	{80.58°, −39.88°, −159.82°}	1.617	{24.75°, 95.71°, 144.35°}	
	OS3	{26.25, −116.20°}	{88.93°, −31.25°, −151.24°}	1.646	{25.13°, 95.05°, 144.96°}	
HRC *r_if_* = 2 mΩ	OS1	{1.89, 114.94°}	{−36.06°, −156.44°, 83.63°}	1.474	{29.12°, 91.38°, 148.69°}	CASI < 1.85, *CADI_hza_* is smallest_,_ HRC in phase-a
	OS2	{5.98, −124.17°}	{80.41°, −39.77°, −159.75°}	1.619	{24.58°, 95.60°, 144.42°}	
	OS3	{10.49, −116.20°}	{88.61°, −31.21°, −151.21°}	1.646	{24.81°, 95.01°, 144.99°}	

**Table 5:** Results of simulation conducted under super-synchronous operating scenarios [OS4, OS5, OS6].

Fault Level	OS Case	Mag. and IPO of FFC in ZSC {*I*_*z* (*A*)_, *θ*_*z* (*deg*_._)_}	IPOs of FFCs in Rotor Currents {*θ_ira_*, *θ_irb_*, *θ_irc(deg_.*_)_}	CASI Value	CADI Values {CASI*_za_*, CASI*_zb_*, CASI*_zc_*}	Diagnostics
0	OS4	{0.06, N/A}	*I_z_* < *I_zth_*			No rotor winding fault detected
	OS5	{0.10, N/A}				
	OS6	{0.36, N/A}				
ITSC *µ* = 0.06	OS4	{29.77, 69.29°}	{−30.41°, 89.68°, −150.62°}	1.886	{38.80°, 158.97°, 81.32°}	CASI > 1.85, *CADI_iza_* is smallest_,_ ITSC in phase-a
	OS5	{44.28, 38.8°}	{−76.53°, 43.02°, 162.26°}	2.708	{37.73°, 81.82°, 201.06°}	
	OS6	{60.24, 26.68°}	{−84.54°, 34.48°, 153.51°}	2.511	{57.66°, 61.16°, 180.19°}	
ITSC *µ* = 0.03	OS4	{10.70, −13.06°}	{−30.49°, 89.61°, −150.48°}	2.063	{43.53°, 76.55°, 163.54°}	CASI > 1.85, *CADI_iza_* is smallest_,_ ITSC in phase-a
	OS5	{14.14, 51.80°}	{−76.98°, 43.04°, 162.69°}	2.495	{24.73°, 94.82°, 214.06°}	
	OS6	{21.13, 35.68°}	{−85.26°, 34.61°, 154.10°}	2.691	{48.86°, 70.16°, 189.19°}	
ITSC *µ* = 0.01	OS4	{2.26, 84.65°}	{−30.48°, 89.57°, −150.50°}	2.725	{54.24°, 174.33°, 56.96°}	CASI > 1.85, *CADI_iza_* is smallest_,_ ITSC in phase-a
	OS5	{1.81, 62.30°}	{−77.09°, 42.96°, 162.89°}	1.928	{14.68°, 105.34°, 224.93°}	
	OS6	{3.34, 43.74°}	{−85.50°, 34.54°, 154.43°}	2.498	{41.52°, 78.35°, 197.84°}	
HRC *r_if_* = 10 mΩ	OS4	{13.04, 67.62°}	{−29.17°, 89.77°, −150.42°}	1.724	{38.45°, 157.39°, 82.80°}	CASI < 1.85, *CADI_hza_* is smallest_,_ HRC in phase-a
	OS5	{21.61, 20.72°}	{−76.70°, 42.75°, 162.71°}	1.767	{55.98°, 63.47°, 183.43°}	
	OS6	{46.92, 57.32°}	{−85.24°, 34.34°, 154.31°}	1.746	{27.92°, 91.66°, 211.63°}	
HRC *r_if_* = 5 mΩ	OS4	{6.52, 67.23°}	{−29.55°, 89.91°, −150.17°}	1.726	{37.68°, 157.14°, 82.94°}	CASI < 1.85, *CADI_hza_* is smallest_,_ HRC in phase-a
	OS5	{10.80, 20.52°}	{−76.88°, 42.84°, 162.81°}	1.772	{56.36°, 63.36°, 183.33°}	
	OS6	{23.46, 57.18°}	{−85.39°, 34.40°, 154.39°}	1.756	{28.29°, 91.58°, 211.57°}	
HRC *r_if_* = 2 mΩ	OS4	{2.61, 67.00°}	{−29.78°, 90.0°, −150.03°}	1.724	{37.22°, 157.00°, 83.03°}	CASI < 1.85, *CADI_hza_* is smallest_,_ HRC in phase-a
	OS5	{4.32, 20.40°}	{−77.0°, 42.89°, 162.88°}	1.775	{56.60°, 63.29°, 183.28°}	
	OS6	{9.38, 57.08°}	{−85.47°, 34.45°, 154.44°}	1.774	{28.39°, 91.53°, 211.52°}	

**Table 6:** Computational burden and latency comparison.

Method	Computational Burden	Latency
Proposed (FFT + CASI + CADI)	Light	Very low
Negative-sequence features	Medium	Low
Park’s vector (modulus/angle)	Medium	Medium
MCSA sidebands (*f_s_* ± *kf_r_*)	High	High

**Table 7:** Detection accuracy comparison under identical scenarios.

Method	Detection Accuracy
Proposed (FFT + CASI + CADI)	97–99%
Negative-sequence features	93–96%
Park’s vector (modulus/angle)	90–94%
MCSA sidebands (*f_s_* ± *kf_r_*)	88–92%

## Data Availability

The original contributions presented in this study are included in the article. Further inquiries can be directed to the corresponding author(s).

## Nomenclature

Acronyms	
DFIG	Doubly fed induction generator
ITSC	Inter-turn short circuit
ZSC	Zero-sequence current
CASI	Cosine Angle Spread Indicator
CADI	Current Angle Difference Indicator
FFT	Fast Fourier transform
FFC	Fundamental frequency component
FFCs	Fundamental frequency components
IPO	Initial phase offset
IPOs	Initial phase offsets
